# Lentisk (*Pistacia lentiscus*) Oil Nanoemulsions Loaded with Levofloxacin: Phytochemical Profiles and Antibiofilm Activity against *Staphylococcus* spp.

**DOI:** 10.3390/pharmaceutics16070927

**Published:** 2024-07-11

**Authors:** Linda Maurizi, Alba Lasalvia, Maria Gioia Fabiano, Eleonora D’Intino, Francesca Del Cioppo, Caterina Fraschetti, Antonello Filippi, Maria Grazia Ammendolia, Antonietta Lucia Conte, Jacopo Forte, Davide Corinti, Maria Elisa Crestoni, Maria Carafa, Carlotta Marianecci, Federica Rinaldi, Catia Longhi

**Affiliations:** 1 Dipartimento di Sanità Pubblica e Malattie Infettive, Sapienza Università di Roma, Piazzale Aldo Moro 5, 00185 Roma, Italy; linda.maurizi@uniroma1.it (L.M.); antoniettalucia.conte@uniroma1.it (A.L.C.); catia.longhi@uniroma1.it (C.L.); 2 Dipartimento di Chimica e Tecnologie del Farmaco, Sapienza Università di Roma, Piazzale Aldo Moro 5, 00185 Roma, Italy; alba.lasalvia@uniroma1.it (A.L.); mariagioia.fabiano@uniroma1.it (M.G.F.); eleonora.dintino@uniroma1.it (E.D.); francesca.delcioppo@uniroma1.it (F.D.C.); caterina.fraschetti@uniroma1.it (C.F.); antonello.filippi@uniroma1.it (A.F.); davide.corinti@uniroma1.it (D.C.); mariaelisa.crestoni@uniroma1.it (M.E.C.); maria.carafa@uniroma1.it (M.C.); carlotta.marianecci@uniroma1.it (C.M.); federica.rinaldi@uniroma1.it (F.R.); 3 Centro Nazionale Tecnologie Innovative in Sanità Pubblica, Istituto Superiore di Sanità, Viale Regina Elena 299, 00161 Roma, Italy; maria.ammendolia@iss.it

**Keywords:** *Pistacia lentiscus* L., bioactive oil, nanoemulsion, antibiofilm activity, *Staphylococcus* spp., mass spectrometry

## Abstract

Most clinical isolates of both *Staphylococcus aureus* and *Staphylococcus epidermidis* show the capacity to adhere to abiotic surfaces and to develop biofilms resulting in a contribution to chronic human skin infections. Antibiotic resistance and poor biofilm penetration are the main causes of ineffective therapeutic treatment in killing bacteria within biofilms. A possible strategy could be represented by drug delivery systems, such as nanoemulsions (composed of bioactive oil, surfactant and water phase), which are useful for enhancing the drug permeation of a loaded drug inside the biofilm and its activity. Phytochemical characterization of *Pistacia lentiscus* oil (LO) by direct infusion Fourier-transform ion cyclotron resonance mass spectrometry (FT-ICR MS) allowed the identification of bioactive compounds with antimicrobial properties, including fatty acids and phenolic compounds. Several monoterpenes and sesquiterpenes have been also detected and confirmed by gas chromatography–mass spectrometric (GC-MS) analysis, together providing a complete metabolomic profiling of LO. In the present study, a nanoemulsion composed of LO has been employed for improving Levofloxacin water solubility. A deep physical–chemical characterization of the nanoemulsion including hydrodynamic diameter, ζ-potential, morphology, entrapment efficiency, stability release and permeation studies was performed. Additionally, the antimicrobial/antibiofilm activity of these preparations was evaluated against reference and clinical *Staphylococcus* spp. strains. In comparison to the free-form antibiotic, the loaded NE nanocarriers exhibited enhanced antimicrobial activity against the sessile forms of *Staphylococcus* spp. strains.

## 1. Introduction

The skin is the body’s largest organ, playing a fundamental role in several physiological processes. It contributes to functions such as vitamin D synthesis, hydration, and regulation of body temperature.

Notably, it serves as a main defense against chemicals and pathogens, with the epidermis acting as the first line of protection [1].

Skin infections are among the most common pathologies found in community and hospital settings. They can range from limited superficial infections, mostly controlled by topical antibiotic treatments, to severe infections of deep tissues that can lead to death if the patient is not appropriately treated [2].

*Staphylococcus aureus*, a versatile bacterium living as a commensal in 30% of people [3], is one of the main causes of human bacterial infections worldwide and the most common agent in skin and soft tissue infections. Due to the emergence of methicillin-resistant isolates, it represents a significant public health problem [4].

At the same way, *S. epidermidis* is a normal inhabitant of the healthy human skin and mucosal microbial communities and has emerged as a common cause of numerous nosocomial infections, mostly occurring in immuno-compromised hosts or patients with implanted medical devices [5].

Most clinical isolates of both *S. aureus* and *S. epidermidis* show the capacity to adhere to abiotic surfaces and to develop biofilms, complex and aggregated communities of microorganisms embedded in a self-producing matrix of extracellular polymeric substances [6]. Antibiotic resistance and poor biofilm penetration are the main causes of ineffective therapeutic treatment in killing bacteria in biofilms [7].

Given this, undoubtedly, this microorganism represents a high-priority pathogen for developing new therapeutic strategies capable of eradicating both planktonic and sessile forms [8]. Among antimicrobial agents, natural products have been extensively explored to avoid the side effects of traditional antibiotics [9,10].

*Pistacia lentiscus* L. is a bush that plays an important role in the ecosystem of the Mediterranean maquis. It is a rustic, drought-resistant evergreen species and has been used for a long time in traditional medicine for the treatment of various kinds of diseases. Almost the whole plant, including the leaves, fruits, wood, and mastic resin, contains a variety of chemical constituents which are medicinally important [11]. Antioxidant, hepatoprotective, anticancer, anthelmintic, wound healing, hypotensive, antiarthritic and antimicrobial properties have been described [12].

Lentisk (*Pistacia lentiscus*) oil (LO) is obtained from *P. lentiscus* berries and is able to promote the repair of skin lesions and inhibit lipid oxidation and depletion of antioxidant defense enzymes [13]. This oil was commonly used in topical treatment of burns, wounds and sores [14] and its antimicrobial activity has been reported [15].

Levofloxacin (LVX) is one of the ofloxacin isomers and a conformationally locked analog of norfloxacin, from the third generation. It was less toxic and more potent than the other dextro form [16]. It is active against Gram-negative and Gram-positive bacteria and possesses significant activity against atypical pathogens, such as *Chlamydia*, *Mycoplasma* and *Legionella* species [17].

LVX is mainly used in respiratory, urinary tract, and soft-tissue infections due to its high renal elimination rate. The marketed products are for parenteral or oral administration and, due to their nearly complete absorption, both formulations may be interchangeable [18]. The fluoroquinolones levofloxacin, gatifloxacin, and moxifloxacin are FDA-approved for both complicated and uncomplicated skin infections. However, due to their broad-spectrum potency, they are commonly reserved for more serious infections. Additionally, levofloxacin is not typically the first choice for MRSA infections due to potential resistance [19].

A recent study reports the inclusion of LVX in nanoparticles as a promising strategy to suppress antibiotic resistance and biofilm formation in *Acinetobacter baumannii* [20].

Furthermore, the combination of nanoencapsulated oregano oil with both ciprofloxacin and gentamicin has been proposed to treat MRSA skin infections [21]. Bastari et al. demonstrated that nafcillin sodium- and levofloxacin-loaded poly (lactide-co-glycolide) nanoparticles coated with calcium phosphate are able to inhibit the formation of an *S. aureus* biofilm [22].

LVX is a lipophilic compound [23] and it can be successfully encapsulated in a bioactive oil phase.

Nanoemulsions (NEs) can be obtained by dispersing an oil phase in water, with droplet sizes on the order of 100 nm; NEs can be easily prepared, and the excipients used for their formulation are generally biocompatible and biodegradable.

Oil-in-water (O/W) NEs have been proposed for cosmetic and drug formulations, notably for lipophilic molecules that can be incorporated and protected in the oily nanodroplets [24]. Additionally, the properties of the oil in the NEs can enhance the activity of the entrapped drug.

Recently, some authors suggested the promising use of NEs against virulence factors in multidrug-resistant *S. aureus* strains [25].

The LO unsaponifiable fraction, predominantly hydrocarbons, monoterpenes and sesquiterpenes, associated with polyunsaturated fatty acids and polyphenols, seems to be the main agent of its regenerative effect at the skin level [26]. Clinical trials have demonstrated the therapeutic potential of Chios mastic. The European Medicines Agency (EMA) acknowledged the resin of *P. lentiscus* L. (mastic) as a traditional herbal medicinal product with two therapeutic indications: mild dyspeptic disorders and skin inflammation/healing of minor wounds. In recent years, Chios mastic has been extensively incorporated into medicinal products, food supplements, and cosmetics, becoming a subject of research in the field of pharmaco-technology [27]. Moreover, several studies have highlighted the anti-inflammatory activity of the essential oil obtained from mastic resin in cases of skin irritation. Furthermore, based on the research on mastic-based liposomes conducted by M. Allaw et al., 2018, PO showed no specific toxicity at precise concentration ensuring the proliferation of keratinocytes and fibroblasts [28]. In our study, due to the wealth of bioactive molecules, especially fatty acids and phenolic compounds, which are key contributors to its numerous beneficial properties, PO could represent an appropriate candidate for developing nanocarrier formulations to improve antimicrobial and antibiofilm activity.

Herein, an untargeted metabolomic approach was performed to achieve a large coverage of phytochemicals in this complex mixture, given the high resolving power and sensitivity of the Fourier-transform ion cyclotron resonance (FT-ICR) mass spectrometer coupled with electrospray ionization (ESI) [29]. This approach allows for a reliable chemical characterization of (semi) polar compounds of LO, thus offering a fast method to perform a qualitative description of the phytochemical fingerprint [30]. Van Krevelen diagrams (vKd) and a donut chart of elemental composition have supported the qualitative analysis by furnishing a graphical description of the metabolomics fingerprint.

The chemical characterization of the LO proposed in this study is an essential preliminary step aimed to quali-quantitatively assess both its primary and secondary metabolome. The volatile and semi-volatile fraction of the seed LO was analyzed through static-HS-GC-MS and HS-SPME-GC-MS with the purpose to subtly determine the fingerprint of this matrix by assigning all the components of the multifaceted terpenes and sesquiterpenes pattern [31].

Moreover, this study aims to design and characterize LO-based NEs, both empty and those loaded with LVX. A deep physical–chemical characterization of LO-based NEs has been carried out to obtain specific nanocarriers suitable to penetrate and efficiently release antibiotic in the thick biofilm. In particular, the characterization study was carried out in terms of hydrodynamic diameter, ζ-potential, morphology, entrapment efficiency, anisotropy, and stability (overtime and at two different storage temperature). In addition, release and permeation (through synthetic membrane) ability were studied. To determine the influence of NEs on bacterial growth, the minimum inhibitory concentration (MIC) and the minimum bactericidal concentration (MBC) were evaluated.

## 2. Materials and Methods

### 2.1. Materials

Lentisk Oil, obtained from cold pressing of *Pistacia lentiscus* berries, was kindly gifted by Soc. Coop. Olivicoltori Valle del Cedrino—Orosei (NU), 08028, Italy, loc. Conculas, S.S. 125 km 225. Brij O10, Diphenylhexatriene (DPH), Levofloxacin, Ethanol, Hepes salt [N-(2-hydroxyethyl)piperazine-N′-(2-ethanesulfonic acid)] were purchased by Sigma-Aldrich (St. Louis, MO, USA). Phosphotungstic acid (PTA) was purchased from Assing S.p.A. (Monterotondo, Rome, Italy).

### 2.2. Mass Spectrometry Analysis

The phytochemical characterization of LO obtained from the mastic tree (*Pistacia lentiscus*) was achieved by means of a multimethodological approach based on both direct infusion Fourier-transform ion cyclotron resonance mass spectrometry (FT-ICR MS) coupled with an electrospray ionization (ESI) source and gas chromatography (GC) coupled with a single quadrupole mass spectrometer. The combination of these two techniques provides high metabolome coverage, revealing a wide spectrum of compounds, from (semi) polar (ESI-FT-ICR MS) to less polar ones (GC-MS). In particular, the ultra-high resolving power, sensitivity and mass accuracy of the FT-ICR MS allows rapid chemical fingerprinting of complex mixtures to be achieved, such as foodstuffs, plant extracts, and essential oils [32]. Herein, a small amount (500 µL) of LO was filtered through a syringe filter (0.45 µm hydrophobic polypropylene, Acrodisc, Sigma-Aldrich, Milan, Italy) to remove particulates and debris. A sample of 250 µL of filtrate solution was diluted in 1 mL methanol/acetonitrile (50/50, *v*/*v*) and vortexed for 3 min; then, the solution was further diluted in the ratio of 1:1000 in methanol, to reach a final 5–10 µM concentration. To enhance ionization, formic acid and ammonia, both at the final concentration of 0.1% (*v*/*v*), were added in positive and negative ionization mode, respectively. The diluted solution was directly infused in the electrospray source hyphenated with a high-resolution mass spectrometer (Bruker BioApex 4.7 T FT-ICR, Bruker Daltonics GmbH, Bremen, Germany). The analyses were performed in both ionization modes (ESI(+), ESI(−)); the syringe flow rate was set at 120 µL/h; the spectra were acquired in the range of 100–1000 *m*/*z*; 200 scans were collected and 3 technical replicates recorded. All raw data were pre-processed on the Bruker Compass DataAnalysis software (3.4 version) for mass calibration and peak picking. The *m*/*z* values with a cut-off S/N of four were submitted to a home-made script for the annotation, wherein each *m*/*z* value was first associated to a univocal molecular formula and then to one or more metabolite isomers. The script has carried out the assignment by enquiring different databases; for each *m*/*z* value, protonated ([M + H]^+^) and alkali metal adducts ([M + Na]^+^, [M + K]^+^), in positive mode, and deprotonated ions ([M − H]^−^) and chloride adducts ([M + Cl]^−^), in negative mode, have been considered. In addition, the analysis revealed the presence of some cationic species that have been detected and indicated as M^+^. A comprehensive list of putative compound annotations, together with theoretical and experimental adduct *m*/*z*, neutral mass, and peak intensity are available in Table S1 (ESI(+)) and Table S2 (ESI(−)). Most of the entries have been annotated considering a mass accuracy lower than ±3 ppm. Collision-Induced Dissociation (CID) experiments have been carried out as additional analyses to confirm the identity of the candidate metabolites by comparing the experimental fragmentation pattern and reference spectra available in the literature or the database. Qualitative and semi-quantitative analyses were furnished by graphical tools, such as van Krevelen diagrams, histograms of relative abundance distribution and donut chart of elemental composition, so giving an overview of the elemental ratio composition and molecular density of several classes of metabolites. Meanwhile, GC-MS is the most suitable technique to assess the Volatile Organic Compound (VOC) profile of a complex matrix, i.e., food, essential oils [33]. Herein, vapors (headspace—HS) in equilibrium with the liquid oil at a controlled temperature were collected through two different approaches. Regardless of the sampling method, a weighted amount of LO (1.8 g) was introduced in a 4 mL vial without any previous pretreatment and let equilibrate with its vapors in a 50 °C thermal bath for 5 min under a stirring speed of 200 rpm (equilibration step). Subsequently, according to the static-HS-GC-MS method, 100 µL of sample HS was drawn into a gas-tight syringe and directly injected into the gas chromatograph. Alternatively, the equilibration step was followed by the solid-phase micro-extraction (SPME) of the sample headspace aimed to selectively preconcentrate the volatile metabolites (extraction step). The medium polarity DVB/CAR/PDMS triple-coated fiber was selected for its wide range of polarity, making it the most suitable fiber for an untargeted analysis [34]. The SPME fiber was exposed to the oil headspace for 50 min at 50 °C under a stirring speed of 200 rpm [35]. HS-SPME method presents a number of advantages over other techniques, being extremely sensitive in unveiling even traces of low- or medium-polarity VOCs crucially contributing to the sensory properties of an edible matrix that, as in the presented case, is proposed to be included in a pharmaceutical preparation. Moreover, the exclusive extraction of VOCs makes this analysis extremely clean and free from solvent contaminants [36]. The gaseous analytes were separated through an Agilent Technologies 6850 gas chromatograph coupled with an Agilent Technologies 5975 mass spectrometer, equipped with the non-polar capillary column HP-5MS (30 m × 0.25 mm inner diameter, film thickness 0.25 µm). The gas-chromatographic parameters were set as follows: inlet temperature, 260 °C; injection mode, split (split ratio 40/1); flow rate of the helium carrier gas (99.995% purity), 1.0 mL/min; oven temperature starting from 40 °C, after 5 min raised to 200 °C at 5 °C/min, and kept at this final temperature for 30 min. Mass spectrometry parameters were set as follows: EI energy, 70 eV; source temperature, 230 °C; quadrupole temperature, 150 °C; and mass scan was carried out in the 50–350 *m*/*z* range. All the analyses were performed in triplicate. Mass and chromatographic data were crossed to support the analytes identification. The experimental EI spectra were compared with those collected in both commercial (FFNSC 3) and free online databases (NIST 11, Flavor2). Kovats index (KI) was then used as the further parameter to confirm the MS-based identification of the analytes. KIs were measured using a mixture of n-alkanes (C7–C40) with the same chromatographic setup, and then compared with values reported in the mentioned database and/or with data reported in the literature. The quantitative analysis was performed automatically, integrating the GC/MS peak areas and calibrated by correction factors that relied on an internal standard.

### 2.3. Ternary Phase Diagram Construction and Nanoemulsion Preparation

The ternary phase diagram, consisting of LO, Brij O10, and Hepes Buffer was constructed. Various prepared mixtures, containing different ratios of oil/water/surfactant are plotted on a two-dimensional horizontal surface, which, by convention, is represented as an equilateral triangle. It is assumed that each vertex and each side represent the percentage, in this case weight/weight (*w*/*w*), of individual components of the emulsion system. At each vertex, there is 100% of one component and 0% of the other two. Every point on the diagram represents a different quantitative composition of the three elements. Suitable quantities of the three components were weighed in a test tube, and then vigorously vortexed for 2 min to ensure complete mixing [37].

For each emulsion, a visual inspection was carried out to classify it as “homogeneous” or “non-homogeneous”. An emulsion was considered homogeneous if there were no distinct particle growth processes occurring and no phase separation appeared, remaining stable. Non-homogeneous dispersions generally appear highly turbid, with evident phase separation of the components.

All the points corresponding to the prepared and inspected formulations are added to the diagram and two areas are delineated on the diagram, whose points correspond to preparations that appeared homogeneous and non-homogeneous to the naked eye.

From the homogeneity region, a sample (NEs A) was selected based on a specific composition regarding the effectiveness and non-toxicity of the oil.

The selected ratio of oil/water/surfactant, reported in Table 1, was vortexed for about 2 min to allow the micro-emulsion to form, and then, the obtained microscale droplets were sonicated for 3 min at 50 °C, using a tapered microtip operating at 20 kHz at an amplitude of 20% (Vibracell-VCX 400, Sonics, Taunton, MA, USA) [38]. At this stage, all formulations can be sterilized by using cellulose filters (0.22 μm) in accordance with Ph. Eur.

The active compound, LVX, was initially dissolved in LO, and the surfactant was then added to the oil/active compound at a weight ratio of 3:1 (*w*/*w*). Hepes buffer (10^−2^ M, pH 7.4) was joined as the aqueous phase, and the resulting mixture was vortexed for approximately two minutes to facilitate the formation of a homogeneous dispersion. The formulation method of LVX-loaded NEs (NEsL) is the same as described previously.

### 2.4. Dynamic Light Scattering and ζ-Potential Measurements

The hydrodynamic diameter and polydispersity index (PDI) of NEs were assessed at 25 °C by using a ZetaSizerNano 90 S (Malvern Instruments Ltd., Worcestershire, UK), equipped with a 5 mW HeNe laser (wavelength λ = 632.8 nm) and a digital logarithmic correlator. Normalized intensity autocorrelation functions were detected at a 90° angle and analyzed using a Contin algorithm to determine the decay time of the electric field autocorrelation functions [39]. By utilizing the decay time, the distribution of the diffusion coefficient D of the particles could be obtained. With the Stokes–Einstein relationship (RH = KBT/6πηD, where KBT represents thermal energy and η represents solvent viscosity), the effective hydrodynamic radius RH was calculated. The reported values of the radii in this study correspond to the intensity-weighted average [40].

The absolute ζ-potential value of the NEs was determined by measuring electrophoretic mobility using the Smoluchowski equation ζ = uη/ϵ, where η and ϵ denote the viscosity and the permittivity of the solvent phase [41].

### 2.5. Oil Droplet Characterization by DPH Fluorescence Anisotropy

Oil droplet characteristics were examined using diphenylhexa-triene (DPH), a fluorescent probe. Specifically, DPH fluorescence anisotropy was utilized to analyze properties of both empty and loaded NEs. DPH-loaded NEs were formulated by co-dissolving the probe in the oil phase at a concentration of 2 × 10^−4^ M, as outlined in prior studies [42]. The fluorescence anisotropy (A) was calculated using the following ratio:

$$A=\frac{\left(I_{vv\;}-I_{vh\;}\right)\times G}{{(I}_{vv}\;+\;{2I}_{vh})\times G}$$

where I_VV_, I_VH_, I_HV_ and I_HH_ are the intensities (λexc = 350 nm, λem = 428 nm) of the fluorescence measured by a LS5013 PerkinElmer (Perkin Elmer, Waltham, MA, USA) spectrophotometer [42], with V (vertical) and H (horizontal) orientation of the polarized light. G = I_HV_/I_HH_ factor is the ratio of sensitivity of the detection system. Results are shown as the average of three different preparations ± standard deviation.

### 2.6. Morphological Analysis

For TEM analysis, both NEs and NEsL dispersions were placed on copper grids and allowed to dry. Grids were counterstained before observation with a 1% aqueous solution of phosphotungstic acid (PTA), adjusted to pH 7.0. The morphology was observed under a FEI 280S transmission electron microscope (FEI Company, Hillsboro, OR, USA) with an accelerating voltage of 100 kV. Image editing was performed by Adobe Photoshop CS4 software (Adobe Systems, San Jose, CA, USA).

### 2.7. Stability Studies

To evaluate NEs colloidal stability over time, both empty and loaded NEs were stored for up to 90 days at two different temperatures (room temperature and 4 °C). Samples were analyzed at specific time points (1, 30, 60, and 90 days) to observe any changes in hydrodynamic diameter and ζ-potential.

### 2.8. Release Studies

In vitro release ability is a fundamental parameter to consider in the formulation and characterization of drug delivery systems. In vitro release of LVX from NEs formulations was investigated using cellulose dialysis tubing (MWCO 8 kDa, Spectra/Por^®^, Rancho Dominguez, CA, USA). The release system was continuously stirred with a magnetic bar (100 rpm) at 32 °C. At predetermined intervals (0 to 8 h, and every 24 h thereafter), an aliquot of the release media was withdrawn, to perform UV analysis, and replaced into the release medium. The absorbance of LVX at λ = 465 nm has been measured using a UV–visible spectrophotometer (LS5013, Perkin Elmer, Waltham, MA, USA).

### 2.9. In Vitro Artificial Skin Permeation Experiments

Strat-M^®^ permeation studies were carried out by using continuous flow Franz-type diffusion cells. This study was performed to evaluate the behavior of NEsL for a hypothetical topical administration.

Vertical Franz cell consists of two compartments: a donor compartment with a capacity of 1 mL, where NEs-L is inserted, and a receptor compartment with a capacity of 5 mL, containing the external medium (Ethanol). An artificial membrane (Strat-M^®^ Millipore, diffusion area of 2.5 cm^2^, purchased from Sigma-Aldrich) is placed between the two compartments to simulate human skin and to estimate the degree of cutaneous permeation of the formulation.

The experiment was conducted at 32 °C to mimic the temperature of the skin in vivo [43].

At predetermined intervals (every hour for 3 h), an aliquot of the medium from the receptor compartment was withdrawn, to perform UV analysis, and replaced into the release medium. At the end of the experiment (after 3 h), the membrane was placed in an ethanol solution to extract the absorbed content from the membrane itself and it was analyzed using a UV spectrophotometer to determine the presence of LVX remaining trapped within the membrane.

In this case as well, the absorbance of LVX at λ = 465 nm has been measured using a UV–visible spectrophotometer (LS5013, Perkin Elmer, Waltham, MA, USA).

### 2.10. Bacterial Strains

*S. aureus* ATCC 6538P and *S. epidermidis* ATCC 35984 were biofilm-forming reference strains obtained from the American Type Culture Collection (ATCC, Manassas, VA, USA). Clinical *S. aureus* isolates were obtained from a collection of strains of the Department of Public Health and Infectious Diseases, “Sapienza” University of Rome. In particular: *S. aureus* HC has been isolated from the anterior nares of a healthy carrier and *S. aureus* DA from the skin lesion of a patient with atopic dermatitis.

The bacterial strains were grown in BHI (Oxoid, Basingstoke Hampshire, UK) and stored in 15% glycerol-BHI at −80 °C.

### 2.11. Determination of Minimum Inhibitory Concentration (MIC) and Minimum Bactericidal Concentration (MBC)

The MIC determination of NEs was performed by the microdilution method and carried out in triplicate. Exponentially growing bacterial cultures were diluted to the cell density corresponding to 0.5 McFarland, and 10 μL of each bacterial suspension was added to 190 µL of BHI (Oxoid, Basingstoke Hampshire, UK) containing the preparations ranged from 2 mg/mL to 0.08 µg/mL. After the incubation at 37 °C for 24 h, MIC was defined as the lowest concentration where there is no visible growth. The inhibition was also evaluated by measuring the optical density (OD) at 595 nm using a microplate reader (Perkin Elmer, Boston, MA, USA).

For the evaluation of MBC, 10 μL from each well with no visible growth were plated on Tryptic Soy Agar (TSA, Oxoid, Basingstoke, UK) for 24 h. MBC is defined as the lowest drug concentration that kills 99.9% or more of the bacterial inoculum.

### 2.12. Bacterial Biofilm Production

Each bacterial strain, at the concentration of 1–2 × 10^6^ CFU/mL, was inoculated into wells of a 96-well polystyrene plate containing Tryptic Soy Broth (TSB Oxoid, Basingstoke Hampshire, UK), added with 1% of glucose, and incubated, as recommended by Stepanovic et al., 2007 [44], for a period of 24 h at 37 °C. The plate was washed with phosphate-buffered saline (PBS, Oxoid, Basingstoke Hampshire, UK) and was allowed to dry. The wells were stained for 15 min with crystal violet (Sigma-Aldrich, 1% *w*/*v*), a basic dye that binds negatively charged molecules. After the incubation time, the dye was solubilized with 95% (*v*/*v*) ethanol for 30 min and the 570 nm OD was measured as described by Stepanovic et al., 2007 [44]. Biofilm production was based on the cut-off OD, which is defined as the three standard deviations above the mean OD of the negative control (ODc). The strains were classified as follows: OD ≤ ODc = no biofilm producers, ODc < OD ≤ (2 × ODc) = weak biofilm producers, (2 × ODc) < OD ≤ (4 × ODc) = moderate biofilm producers, and (4 × ODc) < OD = strong biofilm producers. Uninoculated TSB broth served as a negative control, and experiments were conducted in sextuplicate.

### 2.13. Biofilm Inhibition and Eradication Assay

To measure biofilm inhibition, the growth medium was supplemented with nanocarriers, the free-form antibiotic, or LO at sub-MIC concentrations (0.01 or 0.1 μg/mL). Inhibition was assessed after a 24 h incubation at 37 °C with the substances. Instead, the effect of NEs on pre-formed biofilms was evaluated after 24 h bacterial growth in polystyrene 96-well plates at 37 °C. After this time, the supernatant was removed and sub-MIC concentrations of preparations were added and the plate was incubated for 7 h.

After incubation times, for both inhibition and eradication, the medium was removed, wells were rinsed twice with PBS, and stained with crystal violet (2% *w*/*v*), as previously described. Absorbance was measured at 570 nm with a microplate reader (Bio-rad Benchmark, Hercules, CA, USA). The percentage of biofilm inhibition and eradication was calculated using the following formula [45]:

Biofilm inhibition/eradication (%) = 100 − (OD570 sample/OD570 control × 100)

Values greater than 40% were considered significant. Uninoculated TSB broth served as a negative control, and experiments were conducted in sextuplicate.

### 2.14. Statistical Analysis

Statistical analyses, for antimicrobial studies, were conducted using one-way ANOVA followed by Tukey’s post hoc pairwise tests (GraphPad Prism, Version 5.0). A *p*-value of less than 0.05 (* *p* < 0.05) was considered statistically significant.

## 3. Results and Discussion

### 3.1. High-Resolution Mass Spectrometry Analysis

According to the literature [46], LO holds a large variety of bioactive molecules, in particular fatty acids and phenolic compounds, which are among the main ones responsible for its various beneficial properties, primarily the antimicrobial and antioxidant activities. Results will be presented and discussed below.

**Figure 1:**
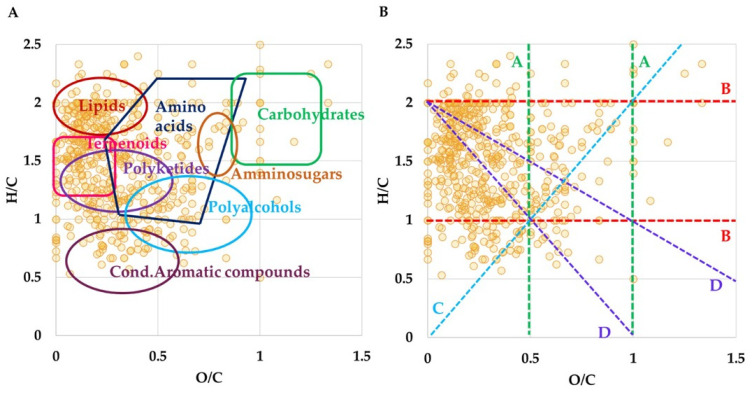
(**A**) Van Krevelen plot gained from the molecular formulas obtained by ESI FT-ICR MS analysis of LO. (**B**) Van Krevelen diagram displays homology series (dashed lines) related to the following chemical reactions: A lines (green) stand for (de)hydrogenation; B lines (red) stand for oxidation or reduction; C lines (light blue) stands for (de)hydration and condensation processes; D lines (purple) stand for (de)methylation.

Overall, the high-resolution mass spectrometric analysis (HRMS) of LO revealed almost 600 univocal molecular formulas, considering both ionization modes. VKd groups the metabolites according to their own O/C versus H/C molar ratio. In the vKd shown in Figure 1A is characterized by a large abundance of lipids, terpenoids and polyketides, followed by a relevant number of amino acids and polyphenols. Furthermore, the vKd has been also used to highlight the metabolic pathways that occur among the entries, triggered by several chemical reactions. In Figure 1B, each group of reactions marks a line: along lines A (green lines) several hits have been found that are related by (de)hydrogenation reactions, e.g., for O/C = 0.28, heptenoic acid (C_7_H_12_O_2_)/heptanoic acid (C_7_H_14_O_2_); for O/C = 0.31, gibberellic acid (C_19_H_22_O_6_)/gibberellin A1 (C_19_H_24_O_6_). Oxidation or reduction reactions are represented by lines B (red lines), along which succinic acid (C_4_H_6_O_4_)/succinaldehyde (C_4_H_6_O_2_) for H/C = 1.50; and cysteine sulfinic acid (C_3_H_7_NO_4_S)/S-oxocysteine (C_3_H_7_NO_3_S) for H/C = 2.33 have been identified. Line C (light blue line), with a slope of 2, shows entries joined by hydratation or condensation reactions; for instance, stearidonic (C_18_H_28_O_2_)/hydroxylinolenic (C_18_H_30_O_3_) acids. Finally, compounds in different alkylation series occur along lines D (purple lines) with an intersect at H/C = 2, including the series of fatty acids, such as myristic (C_14_H_28_O_2_)/palmitic (C_16_H_32_O_2_)/stearic (C_18_H_36_O_2_)/arachidic (C_20_H_40_O_2_) acids.

**Figure 2:**
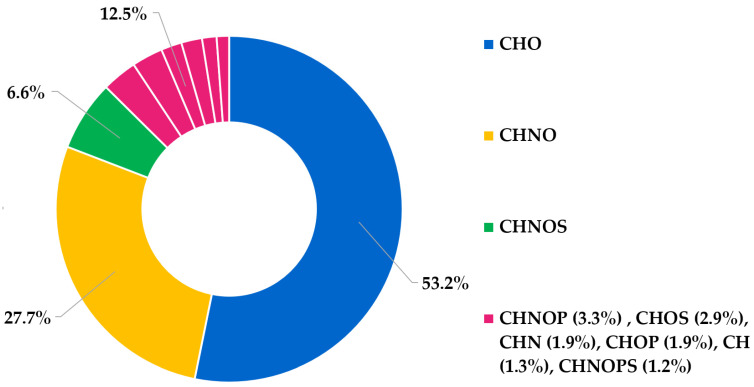
Donut chart regarding the elemental composition of LO sample. The chart shows the relative frequency of the CHO, CHNO, CHNOS, CHNOP, CHOS, CHN, CHOP, CHNOPS population.

Moreover, the donut chart of elemental composition (Figure 2) reports the percentage of different entries identified by the MS analysis. The graph establishes CHO as the most abundant class of compounds (>50%), mainly represented by fatty acids, polyphenols and terpenoids, followed by CHNO (ca. 28%), including entries such as amino acids, dipeptides, and amides of fatty acids, and by CHNOS (ca. 7%). All other molecular classes (CH, CHN, CHNOP, CHNOPS, CHOP, CHOS) contribute less than 5% each.

**Figure 3:**
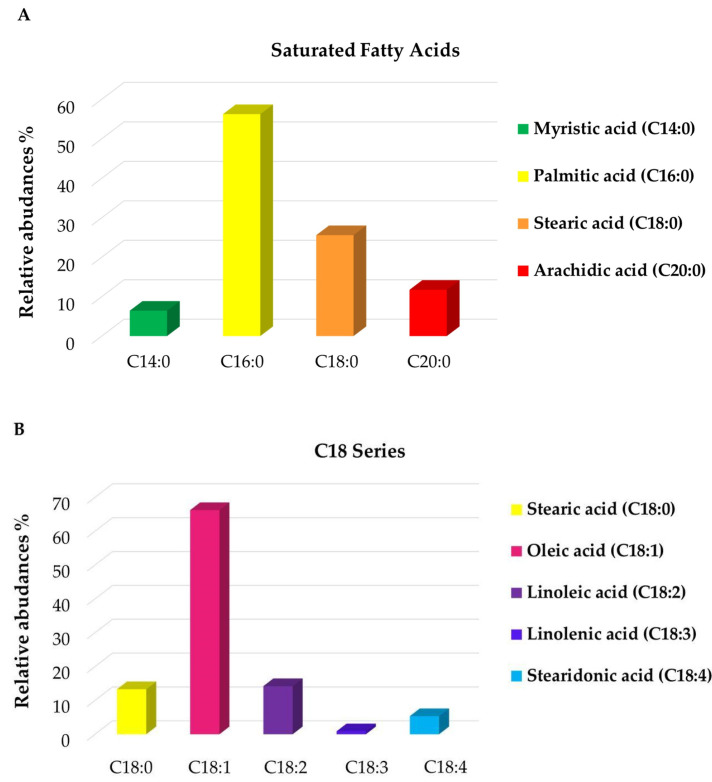
Histograms of the relative abundance distribution within specific classes of FA: saturated (**A**); C18 series (**B**) obtained by ESI(−) FT-ICR MS analyses of LO sample.

Direct infusion ESI FT-ICR MS is a powerful method with the ability to provide a comprehensive description of the composition of complex mixtures, through the identification of a huge number of metabolites by a single and fast measurement. Despite this, an accurate quantitative analysis is not feasible, mainly due to possible ion suppression phenomena and different ionization responses of the numerous components present in natural mixtures, thus making the quantification challenging. However, these effects may be neglected for metabolites that belong to the same class, thus showing similar chemico-physical characteristics. As previously reported [47], the molecular formulas and the abundance of lipids, extracted from mass list gained by ESI(−) MS analysis, have been summed up here in two main clusters: saturated medium-long chain fatty acids (SFA, 18:0–20:0) and C18 series (18:0–18:4) (Figure 3A,B). Figure 3A displays that the most abundant SFA are palmitic acid (C16:0) and stearic acid (C18:0), reaching 56% and 26%, respectively, followed by arachidic (12%) and myristic (6%) acids. Notably, considering the unsaturation degree within the C18 series, the percentage of oleic acid (C18:1) appears relatively higher (>60%), followed by stearic and linoleic (C18:2) acids that both achieve ca. 15%. Linolenic (C18:3) and stearidonic acid (C18:4) turned out to be the least abundant fatty acids, with values less than 1% and 5%, respectively. Thereby, FT-ICR MS analysis has confirmed a composition rich in saturated and unsaturated fatty acids, particularly palmitic (C16:0) and oleic (C18:1) acids. These molecules, along with capric (C10:0), linoleic (C18:2) and hydroxylinoleic acids, all identified in the analyzed sample, are well recognized for their antimicrobial properties, in particular their activity against *S. aureus* and *S. epidermidis* [48]. Indeed, as reported in the literature [49], long-chain unsaturated fatty acids, including some of the fatty acids identified herein, exhibit their antimicrobial property by inhibiting bacterial enoyl-acyl carrier protein reductase (FabI), consequently stopping the bacterial fatty acids’ biosynthesis. In addition, palmitoleic acid (C16:1), found in ESI(−) MS at *m*/*z* 253.21685 as a deprotonated ion, is endowed with selective antibacterial activity against *S. aureus* [50].

Numerous phenolic compounds, such as organic acids, terpenes and flavonoids, have been also revealed. Due to the presence of hydroxyl groups, phenolic compounds show high antimicrobial activity by disrupting cellular membrane integrity [51]. Among the organic acids, to name a few, chlorogenic acid (5-caffeoylquinic acid), vanillic acid, a hydroxybenzoic acid, hydroxycinnamics acids, like p-coumaric, caffeic, feruloylquinic and dihydroferulic acids, and an intermediate in the metabolism of ferulic acid, were found. These metabolites refer to the general families of chlorogenic acids known for their antioxidant, anti-inflammatory and anticancer properties [52]. In particular, chlorogenic acid acts as an antimicrobial agent by irreversibly permeating the bacterial cell membrane, leading to an alteration of intracellular potential and consequent cell death [53]. ESI FT-ICR MS revealed the presence of a rich variety of terpenes and terpenoids, endowed with antioxidant and antimicrobial activities [54], like monoterpenes as myrcene [C_10_H_16_ + H]^+^ discovered at *m*/*z* 137.13247 and also confirmed by GC-MS, monoterpenoids, such as geraniol [C_10_H_18_O_4_ + H]^+^ revealed at *m*/*z* 203.12755, citronellic acid, limonene aldehyde, and thymol/carvacrol (C_10_H_14_H) isomers identified at *m*/*z* 149.09815 as deprotonated ion, and at *m*/*z* 189.06837 as potassiated adduct. As reported in recent works [55], the latter complex is known for the antimicrobial activity alongside the potential reduction in biofilm formation against *S. aureus.* MS analysis also evidenced the presence of sesquiterpenes, including gingerol and cadalene, revealed as protonated species. Furthermore, geranyl-diphosphate, an intermediate in the biosynthesis pathway of terpenes, with a toxic effect in vitro on *E. coli* [56], has been identified at *m*/*z* 315.07614 ([M + H]^+^). ESI FT-ICR MS investigations has also highlighted the presence of numerous flavonoids, flavans, and their derivates in the form of aglycones, such as apigenin (flavone) and myricatin (galloyl flavanonol sulfate), or glycosides, e.g., myricetin 3-(3″,4″-diacetylrhamnoside), quercetin 3-(6″-malonyl-glucoside), quercetin 3-O-xylosyl-glucuronide, luteolin 7-O-(6″-O-malonyl)-beta-D-diglucoside, kaempferol 3-O-arabinoside. The general class of flavonoids, phenolic secondary plants metabolites, holds a wide range of potential activities. They have been thoroughly studied for the selective antibacterial activity and synergism with antibiotics on several bacterial strains. As reported in the literature [57], flavonoids display direct antibacterial activity and suppression of bacterial virulence. To explain these effects, many mechanisms have been proposed, including cytoplasmic membrane damage, inhibition of nucleic acids synthesis and inhibition of energy metabolism. In addition, it has also been described that flavonoids exhibit synergism with antibiotics by both improving β-lactam resistance and enhancing their efficacy in β-lactamase inhibition. Here, both ESI(+) and ESI(−) ionization modes have unveiled the presence of several water-soluble vitamins, like folic acid (vitamin B9), and fat-soluble ones, including vitamin D derivates (hydroxycalcitriol), vitamin A, vitamin K and two isoforms of vitamin E, hydroxy-gamma-tocotrienol and alpha-tocotrienol, acknowledged for their antioxidant activity [58]. During the MS experiments, numerous organosulfur compounds have emerged. All these substances are bioactive metabolites typical of Brassicaceae (sulforaphane) and Liliaceae (allyl sulfide, garlicin, S-Propyl-L-cysteine and S-(Allylthio)-L-cysteine) families, although they are very common in many plant sources. S-compounds present different health properties, including antimicrobial, antioxidant, and anticancer activities [59]. However, the overall beneficial activity can be attributed to the whole phytocomplex present in LO since several phytochemicals, reported above, may contribute with their additive and synergistic effects to the bioactive properties of LO. Interestingly, HRMS analysis revealed the presence of resmetrhin identified at *m*/*z* 377.15113 as potassium adduct [C_22_H_26_O_3_ + K]^+^, an insecticide used in the control of mosquito population.

### 3.2. Static HS and HS-SPME-GC-MS Analysis of the VOC Profile

**Figure 4:**
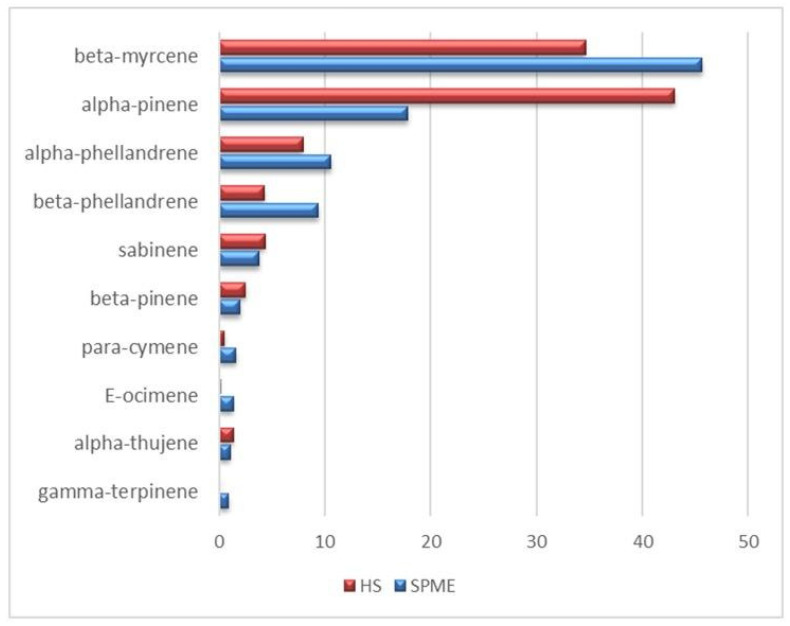
Percentage areas of the most concentrated monoterpenes detected through static HS (red bars) and HS-SPME-GC-MS (blue bars) analyses.

**Figure 5:**
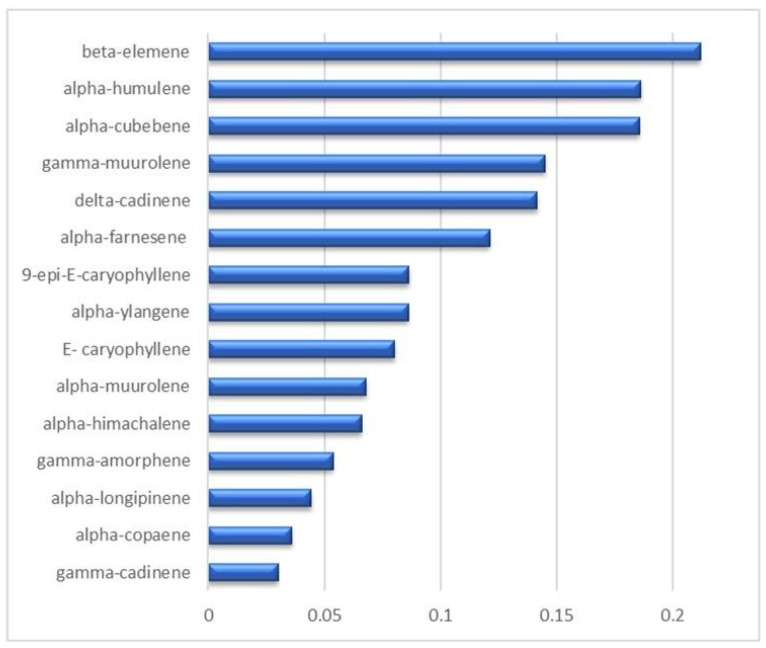
Percentage areas of the sesquiterpenes detected through HS-SPME-GC-MS analyses.

The static HS-GC-MS analysis allowed the exclusive detection of the monoterpenes fraction (Table S3 in Supplementary Materials), that was disentangled providing a detailed VOC profile of the analyzed LO with the relevant percentage composition. In substantial agreement with the literature [60], the most significant components (red bars in Figure 4) are alpha-pinene (43.1%), beta-myrcene (34.6%), alpha- and beta-phellandrene (7.9 and 4.3%, respectively), and finally sabinene (4.4%). All the other monoterpenes, detected in traces (<0.5%), are listed in Table S3. The presence of the monoterpenes class of compounds encourages the potential application of a formulation LO-based as topical agent, since its involvement in the dermal re-epithelialization process has already been pointed out [61]. Static HS sampling proved to be extremely useful to take the actual snapshot of the vapor phase in equilibrium with the liquid oil at a temperature which is selected as a compromise between the maximization of the analytic efficiency and the limit of the analytes thermolability. Complementarily, the exposure of a DVB/CAR/PDMS fiber into the oil headspace, i.e., HS-SPME-GC-MS, provided a preconcentration of the vapor phase which is mandatory to reveal less volatile and/or more diluted compounds. The emerging picture is necessarily more informative than the static HS one because, on the methodological standpoint, the selectivity of the fiber-coating introduced a bias in the headspace fingerprinting, but provided the identification and quantification of metabolites that were invisible to other techniques. Indeed, the HS-SPME-GC-MS technique allowed to detect 47 compounds (Table S4), mostly represented by monoterpenes (96%), according to the VOC profile that emerged in the static HS analysis, that exhibited a definite lower sensitivity (13 detected compounds). Within this class of VOC, significant differences were found in the static HS vs. HS-SPME distributions, as reported in Figure 4, where there is a clear inversion of the myrcene/alpha-pinene ratios (0.8 and 2.6, respectively) indicating a higher affinity of the fiber coating toward myrcene. A further contribution to the VOCs profile emerging from the HS-SPME sampling consists of sesquiterpene detection (Figure 5 and Table S4). In addition to their low relative abundance (17 compounds, about 1.5% of the total chromatogram area), sesquiterpenes represent the second most abundant class of compounds detected in the HS-SPME-GC-MS analysis. Beta-elemene, alpha-humulene and alpha-cubebene are the most representative sesquiterpenes in the LO, followed by gamma-muurolene-alpha-farnesene, 9-epi-E-caryophyllene and its diastereoisomer E-caryophyllene. The antifungal and anti-bacterial activity of sesquiterpenes was extensively reported by Li et al. [62]. Very low abundance (<0.1%) of aldehydes (pentanal and hexanal), ketones (heptyl-methyl-ketone and undecan-2-one) and FAE (isobutyl- and isopentyl hexanoate and methyl-decanoate) were also detected (Table S4). The comparison between the two sampling procedures herein proposed points to their strict complementarity that provides both the actual composition and the selectively enhanced profile of the VOCs.

### 3.3. NEs Design and Physicochemical Characterization

**Figure 6:**
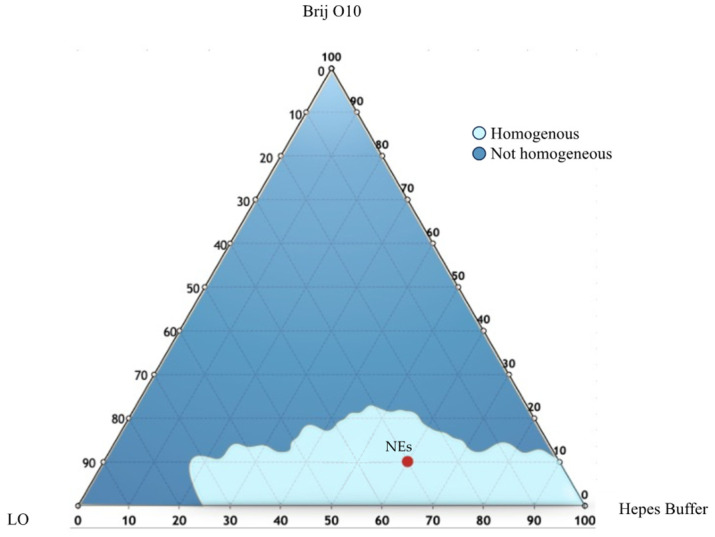
Ternary phase diagram of LO, Brij O10, and Hepes Buffer. The resulting phases were the homogeneous phase (light blue area) and the not homogeneous phase (dark blue area). NEs composition has been placed into the homogeneous region.

To select the appropriate NEs in terms of composition, a ternary phase diagram of LO, Brij O10 and Hepes buffer was developed. Several formulations were prepared with different amounts of each component. The samples were analyzed by visual inspection to identify homogeneous dispersions or not homogeneous ones. These results have been useful to ternary phase diagram construction (Figure 6), in which the light blue region represents the homogenous region, and the blue zone is the non-homogenous one.

To prepare NEs with suitable physical–chemical features, all samples of the light blue region area were sonicated (under the conditions described in Section 2). The selection was made taking into consideration several parameters such as hydrodynamic diameter and ζ-potential. The composition of the chosen sample (NEs) has been detailed in Table 2 and its features (DLS results and anisotropy) outlined in Table 2.

In particular, NEs were characterized by a hydrodynamic diameter of 165.7 nm and a size distribution that was nearly monodisperse (PDI ≤ 0.3) [63].

**Figure 7:**
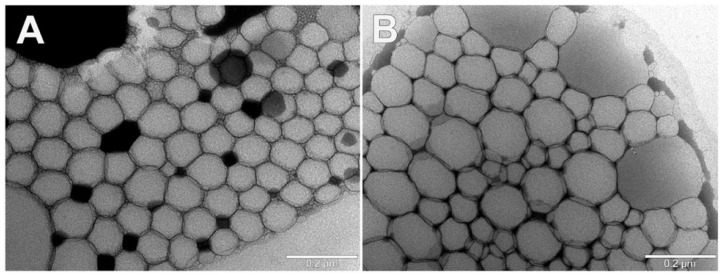
Representative images of the transmission electron microscopy observations of selected empty NEs (**A**) and NEsL (**B**) samples.

Visual inspection of selected NEs by TEM revealed that both NEs and NEsL samples were spherical vesicles with dimensions comparable to DLS measurements. Empty NEs showed tighter aggregation than NEsL due to the droplet dehydration effect due to the TEM procedure, and appeared with smaller size than LVX-loaded vesicles (Figure 7A). Furthermore, LVX loading does not appear to be able to modify the size and ultrastructure of the NEs (Figure 7B).

The E.E. % value of LVX NEs (NEsL) is shown in Table 2. First of all, it is observable that the EE% value of the sample is 100%, suggesting the oil capability to solubilize the drug [64]. Therefore, it is possible to affirm that NEs formulation is able to greatly improve the water solubility of this hydrophobic molecule. Moreover, the drug inclusion inside the nanocarriers does not affect the size and PDI values with respect to empty NEs (Table 2). Both formulations were characterized by similar negative ζ-potential values able to prevent vesicle aggregation during the storage due to the superficial electrostatic repulsion. In fact, according to Moraru et al., 2020 [65], a ζ-potential value bigger than ±20 mV assures sample stability. Furthermore, ζ-potential contribution could also affect drug delivery performance. In fact, as described by Ferreira et al., 2021 negative values of ζ-potential could ensure better interaction and penetration within the bacterial biofilm, due to their capability to prolong the NEs/biofilm interaction time [66].

The oil droplet rigidity plays a critical role in the retention and release of the loaded drug in the nanocarriers, and the rigidity also affects the in vivo circulation time and the interaction capability of NEs with membranes or cells [67]. DPH experiments allow to collect information on oil droplet anisotropy that is correlated with the rigidity of the lipophilic compartment of the nanocarrier. By fluorescence characterization, anisotropy values have been obtained. Due to the hydrophobic nature of DPH, it was localized into the oil core of the NEs. The low anisotropy value indicates that the probe rotational diffusion is not hindered, which can indicate that the fluidity of the local environment is elevated [68]. Both samples, empty and loaded NEs, showed a low and similar anisotropy value (0.24 for empty NEs and 0.26 for loaded ones) and this result suggests a quite fluid oil droplet that could assure a significant release ability of a loaded drug [24].

### 3.4. Physicochemical Stability

**Figure 8:**
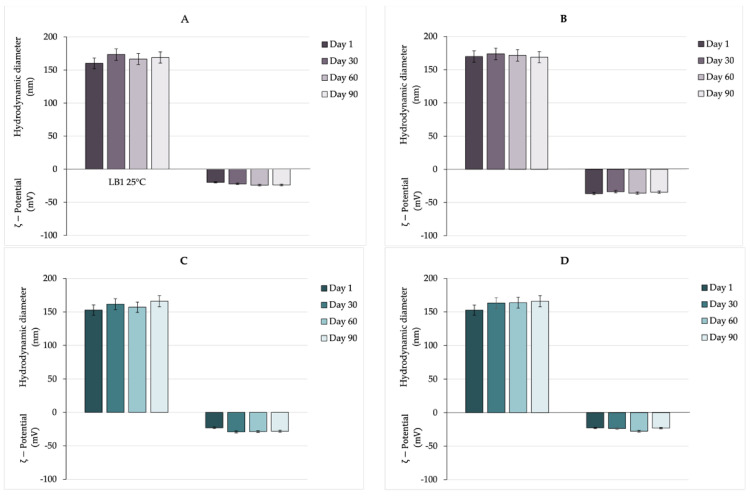
Stability study over time of NEs and NEsL at room temperature (**A**,**C**) and 4 °C (**B**,**D**).

Stability is the crucial parameter of any nanocarrier in which several aspects must be understood carefully to produce a future commercial product. During the design and the first steps of the production of the nanocarrier, various factors must be considered to obtain a stable nanocarrier [69]. The preparation method, such as the component choice, should be selected to optimize droplet size distribution since it strongly affects stability behavior. In particular, regarding composition, the surfactant employed, its concentration, and oil type, are of great importance [70]. Moreover, the hydrodynamic diameter (i.e., systems with droplets diameter smaller than 200 nm usually have a homogeneous and stable structure) together with an opportune ζ-potential value (able to preserve the coalescence phenomena) could affect the stability of the nanocarriers [71]. The stability of nanocarriers is determined by the balance between electrostatic repulsion due to their surface charge and short-range attractive interactions between nanoparticles [72]. Ζ-potential plays a crucial role in the stability of formulated NEs. As reported in the literature, high negative values of ζ-potential prevent aggregation or precipitation phenomena [73]. In an ideal scenario, nanocarriers need to remain stable, avoid aggregation, and retain the drug until they reach the target site. Instability can negatively impact their effectiveness in vivo. Consequently, evaluating the stability of nanocarriers is a crucial aspect of their characterization. Various techniques exist to predict the stability of nanocarriers in different environments—physical, chemical, and physiological—depending on the specific nature of the nanocarriers. Freezing and lyophilization are widely employed for the long-term storage of several nanoparticles. Nevertheless, these processes can induce stresses from crystallization and vacuum dehydration that may damage macromolecules, reducing the stability of the nanoparticles. To mitigate this issue, suitable cryoprotectants could be employed but the added components could affect the drug carrier’s features [74]. In the present study, the nanocarriers’ stability in aqueous condition has been evaluated. To assess the stability over time of empty and loaded NEs, all the samples were stored at room temperature and 4 °C for 90 days and hydrodynamic diameter and ζ-potential were evaluated. Analyzing the obtained results reported in Figure 8, it is possible to affirm that all samples preserved their size and ζ-potential at both 4 °C and room temperature for up to 90 days, with only a slight and not significant increase of a few nanometers in particle size observed in both empty and loaded NEs. Probably, the samples are characterized by a significant stability over time due to (i) negative ζ-potential values (−24 mV/−23 mV, respectively, for empty and loaded NEs) representing the ability to prevent aggregation or precipitation phenomena; (ii) dimensions smaller than 200 nm; and (iii) appropriate preparation method (high-energy method, with specific and selected sonication parameters) able to control NEs droplet size [75]. These results suggest that an aqueous condition is a suitable way for long-term storage of NEs.

### 3.5. In Vitro Release Studies and Permeability Studies

To evaluate the NEs capability to release the entrapped LVX, release studies were performed. In vitro release experiments are commonly employed to predict in vivo behavior, historically with traditional dosage forms such as capsules and tablets (following the dissolution), and more recently with novel dosage forms like injectable, biodegradable, nanocarriers, microspheres, and implants [76].

**Figure 9:**
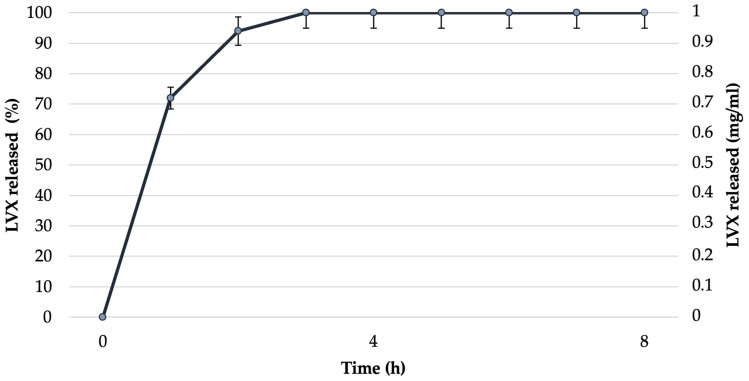
LVX release profile from NEsL by cellulose dialysis tubing.

In vitro release studies have been performed at 32 °C (to mimic the physiological temperature). In particular, Figure 9 shows the concentration versus time profiles of LVX released by NEsL during the experiment by cellulose dialysis tubing. The results obtained demonstrate that the sample was characterized by a high LVX percent release (almost 100% in 3 h) probably due to the oil nanodroplet fluidity investigated by anisotropy fluorescence study.

**Figure 10:**
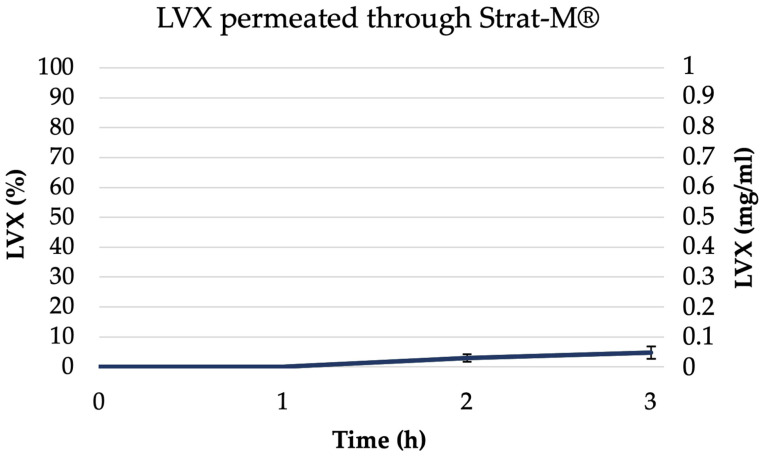
Permeated amount of LVX from NEsL through Strat-M^®^ in a Franz cell system.

In vitro diffusive models are crucial tools for screening the penetration of active compounds from various formulations, helping to predict the success of dermal or transdermal therapies [77]. The standard technique for studying in vitro permeation of drugs involves using a Franz diffusion cell device along with a Strat-M^®^ artificial membrane [78]. Strat-M® is a synthetic, non-animal-based membrane model useful to predict drug permeation capability through human skin. Its use eliminates lot-to-lot variability and addresses common issues related to safety and storage. The membranes are composed of 300 μm thick polyethersulfone and showed a good agreement in the permeability data (compared with human skin), which allows it to be used as a surrogate for human skin. Therefore, the amount of permeated drug per unit area of Strat-M^®^ after 3 h from NEsL was investigated using Franz diffusion cells and was presented in Figure 10. The figure showed the amount of LVX (% and concentration mg/mL) in the acceptor compartment during the experiment (described in Section 2). Probably, the NEs features (together with specific surfactant concentration and drug characteristics) could directly influence the drug permeation through the Strat-M^®^membrane resulting in non-significant levels of LVX in the acceptor compartment [79]. In addition, the permeation study conducted for 24 h has been performed and, according to the results reported in Figure S1, it is possible to observe that no significant increase in LVX permeation has been detected. In conclusion, Strat-M^®^ diffusion experiments suggest that the NEsL could be able, after in vivo administration, to interact only with the biofilm of bacteria and not induce transdermal activity.

### 3.6. Antibacterial Activity

The MIC and MBC of these preparations were evaluated through broth microdilution assay, bringing scalar concentrations of the substances into contact with the bacterial strains as described above.

The antimicrobial activity of edible oil obtained from the berries of *P. lentiscus* has been demonstrated [19]. In our experiments, notwithstanding the most abundant SFA in oil, were palmitic acid and stearic acid, which have shown remarkable antibacterial properties towards Gram-positive and Gram-negative bacteria [80]; LO alone was not able to inhibit bacterial growth, in accordance with those obtained from Orrù G. et al., 2017 [15], who did not appreciate the *S. aureus* strains’ sensitivity to *P. lentiscus* berry oil.

Divergent results regarding levels of antimicrobial activity were reported in the studies testing LO and their extracts. For example, a high antimicrobial activity was demonstrated in regard to the leaves from plants growing in different regions, whereas the fruits from Tunisia and Sardinia showed a limited effect against bacteria and yeasts [81]. These differences were explained by some factors such as the oil’s geographic origin, climatic conditions, and soil composition and plant nutrition [81].

When planktonic staphylococci were assayed for LVX susceptibility, each strain showed a peculiar antimicrobial activity. Interestingly, bacterial strains did not demonstrate appreciable sensitivity to empty NEs but the profiles changed when these nanocarriers were loaded with the antibiotic (Table 3). In fact, when bacterial strains were incubated with NEsL for 24 h, similar MIC and MBC values, with respect to free LVX, were detectable. These results are consistent with numerous studies that have discovered MIC and MBC values either equivalent to or much higher than those of their free-form antibiotics [82]. As reported by the authors, one possible explanation was the incomplete release of antibiotics from nano-antibiotics, resulting in an insufficient number of free antibiotics that are able to effectively exert their action.

Our observation, showing retained MIC values with respect to LVX alone, led us to suppose the ability of NEsL to release antibiotics in adequate quantities in the bacterial growth medium and at the same time carefully preserve its activity.

### 3.7. Biofilm Inhibition and Eradication Ability

In our study, the strain biofilm production ability was classified as reported by Stepanovic et al., 2007 [44] in no biofilm, weak, moderate, and strong producers. The results obtained showed that clinical and, as expected, reference strains [83] were strong biofilm producers.

To verify the efficacy in preventing bacterial biofilm, sub-inhibiting concentrations (0.01 or 0.1 μg/mL) of nanocarriers, the free-form antibiotic, or LO were put in contact during biofilm formation for 24 h.

As shown in Table 4, when the preparations were incubated during the bacterial biofilm formation step, all failed to inhibit its production by *Staphylococcus* spp. isolates. Interestingly, only LVX repressed, in a significant manner, the amount of biofilm produced by the *S. epidermidis* strain. Several studies have reported the antibiofilm activity of LVX, against Gram-positive and Gram-negative bacteria, but often in association with other compounds [84]. Recently, to combat surface-adherent bacteria and potential biofilm formation by *S. epidermidis*, a method has been developed to impregnate LVX antibiotic into the outer polymer of the catheter [85].

The effect on pre-formed biofilms was evaluated after 7 h of incubation with nanocarriers, the free-form antibiotic, or LO at sub-MIC concentrations (0.01 or 0.1 μg/mL).

In our research, the low sub-inhibiting concentration of LVX (0.01 and 0.1 μg/mL) failed to act against *Staphylococcus* spp. reference strains in sessile form even if the antibiotic was loaded in nanocarriers. Interestingly, at the same concentration of 0.1 μg/mL an ameliorate, but non-significant, activity of both LVX and LO was observed against both clinical strains.

It has been reported that fluoroquinolones are able to eradicate biofilms in vitro [86]. Recently, Mazzantini et al., 2024, demonstrated that free LVX remove *S. aureus* ATCC 6538P and *S. epidermidis* ATCC 35984 pre-formed biofilm in a dose-dependent manner [87] at about 8 and 256 μg/mL, respectively. Therefore, our results could be attributed to lower concentrations used in our experiments, unable to produce an inhibitory action.

The most prominent eradicating effect was obtained against the biofilms formed by clinical *S. aureus* DA and *S. aureus* HC with percentages of eradication of 71.0 ± 10.0 and 41.0 ± 0.3, respectively, by NEs loaded with LVX.

Noteworthily, NEs showed eradicating ability against both *S. aureus* HC and, even if in a not significant manner, against *S. epidermidis* strains which could suggest a potential action by the LO. As suggested by other authors, LO might be able to contrast the formation of pathogenic biofilm [19] but in addition, in the NE formulation, preserve and, above all, strengthen biological activities.

As suggested by Ryan et al., 2018 [88], oil-loaded nanoemulsions could exhibit excellent biofilm penetration ability owing to the enhanced interactions between the nanocarrier surface and biofilm matrices compared to hydrophobic oils alone. Recently, Park et al., 2023, demonstrated the activity of pseudopyronine analog-loaded nanoemulsions against preformed sessile forms of *S. aureus*, suggesting the facilitated solubilization and effective delivery of the polymer deep into the biofilm matrix [89]. Anderl et al., 2000 reported that resistance to LVX cannot be attributed to its low diffusion, but rather to its failure to kill bacteria in the biofilm [90]. In this regard, it is necessary to underline that biofilm-related antimicrobial resistance is partly due to the presence of some dormant *S. aureus* cells, also known as persister cells. These cells maintain their dormancy condition during antimicrobial treatment and become active again as soon as the treatment is withdrawn, thus causing a chronic recurrent infection [91]. As already demonstrated for *Pseudomonas aeruginosa*, it could be supposed that the overall density achieved by nanoemulsions can be quite high within the biofilm. Furthermore, the continued release of the antibiotic from biofilm-localized nanostructures could provide a sustained level of the antibiotic near persister cells. It is conceivable that this subpopulation may be better addressed by antibiotic-loaded nanocarriers compared to free drugs [92].

## 4. Conclusions

The conventional treatments for *Staphylococcus* spp. infections mainly involve the use of antibiotics. Some bacterial strains have become resistant to almost all commonly available antibiotics and some kinds of disinfectants [93]. Chronic infections, particularly those involving biofilms, pose a significant challenge in clinical settings. Nowadays, nanomaterial approaches, able to interfere with bacterial adherence, colonization, and biofilm formation, represent emerging alternatives to address these challenges [94].

The mass spectrometry approach, involving the combined application of ESI FT-ICR-MS and GC-MS, led to a quali-quantitative assessment of the (semi)polar and volatile fractions of LO. Phytochemical profiling has revealed the presence of numerous metabolites recognized for their antimicrobial, antibacterial and antibiofilm activities. In particular, palmitic and oleic acids, along with stearic and linoleic acids, were found to be the most abundant fatty acids in LO. Moreover, a wide range of secondary metabolites have been identified, including flavonoids, which are largely studied for their synergism with antibiotics; monoterpenes, primarily represented by alpha-pinene and beta-myrcene; and sesquiterpenes, mainly depicted by beta-elemene, alpha-humulene and alpha-cubebene. This rich composition in bioactive compounds makes LO a potential antimicrobial/antibiofilm agent useful as an adjuvant in topical formulations against bacterial strains.

The use of an oil-based nanocarrier, combined with antibiofilm agents, represents a promising strategy to enhance the efficacy of topical treatments for these persistent infections. In this context, our study using an antibiotic with high susceptibility rates and anti-staphylococcal activity, loaded into NEs, can serve as a starting point for developing new strategies that are currently under investigation. NEsLs have been thoroughly characterized, and it should be emphasized that they exhibited suitable physical–chemical characteristics for the proposed application, and interesting antibiofilm effects against clinical strains at low sub-inhibiting concentrations. Thus, a reduction in the antibiotic dose required for treatment could be achieved, leading to a decrease in side effects.

In this context, further studies are required to determine the following: (i) the cytotoxicity of these preparations and (ii) the optimal semisolid topical formulation capable of incorporating loaded nanoemulsions for in vivo skin disease treatment. Furthermore, although the microtiter plate is a versatile and easy-to-use biofilm reactor that exhibits good reproducibility [95], and the crystal violet staining assay is a method extensively applied in biofilm studies, the present results can be influenced by minor experiment variation [96]. Further studies are planned to establish the origin of the high variability observed in our results and to explore the molecular mechanisms involved in the eradication process. However, despite the extensive ongoing research, only a few of the promising preclinical studies have translated into clinical trials [97]. Our preliminary study could represent a starting point for future treatment opportunities against *S. aureus* infections using the innovative tools of nanomedicine.

## Figures and Tables

**Table 1:** Sample composition.

Sample	LO (mg/mL)	Brij O10 (mg/mL)	LVX (mg/mL)
NEs	0.3	0.1	-
NEsL	0.3	0.1	1.0

**Table 2:** Physico-chemical characterization of samples in terms of hydrodynamic diameter, polydispersity index (PDI), ζ-potential, LVX entrapment efficiency percentage (EE %), and anisotropy (fluidity).

	Hydrodynamic Diameter ± SD (nm)	PDI ± SD	ζ-Potential ± SD (mV)	EE (%)	Anisotropy A.U. (Fluidity)
NEs	165.7 ± 2.5	0.22 ± 0.02	−24.3 ± 0.2	-	0.24
NEsL	152.6 ± 2.9	0.21 ± 0.01	−22.9 ± 1.2	100	0.26

**Table 3:** MIC and MBC values of NEs, NEsL and LVX.

	MIC (µg/mL)			MBC (µg/mL)		
	NEs	NEsL	LVX	NEs	NEsL	LVX
*S. aureus* ATCC 6538P	2000	0.08	0.08	2000	0.08	0.08
*S. aureus* DA	2000	0.63	0.63	2000	0.63	0.63
*S. aureus* HC	2000	0.31	0.08	2000	0.31	0.08
*S. epidermidis* ATCC 35984	1000	0.31	0.31	1000	0.63	0.31

**Table 4:** Biofilm inhibition and eradication ability of NEs, NEsL, LVX and LO. All substances were used at 0.1 μg/mL, with the exception of the conditions with * that were tested at 0.01 μg/mL because the MIC value was lower. Values > than 40% were considered significant in inhibition and eradication of biofilm.

	Biofilm Inhibition				Biofilm Eradication			
Strains	NEs	NEsL	LVX	LO	NEs	NEsL	LVX	LO
*S. aureus* ATCC 6538P	0.3 ± 1.3 *	14.0 ± 12.8 *	0.6 ± 1.0	14.7 ± 2.9 *	24.4 ± 2.9 *	3.2 ± 3.0 *	16.0 ± 3.5 *	4.5 ± 2.1
*S. aureus* DA	25.0 ± 2.5	36.25± 12.3	24.7 ± 22.0	4.75 ± 9.5	21.0 ± 15.6	71.0 ± 10.0	30.8 ± 3.2	31.0 ± 4.3
*S. aureus* HC	24.1 ± 5.6	8.0 ± 6.0 *	23.0 ± 9.5	19.5 ± 6.3	40.0 ± 17.0	41.0 ± 0.3	33.7 ± 2.0 *	33.0 ± 4.8
*S. epidermidis* ATCC 35984	0.2 ± 1.5	30.0 ± 5.0	56.0 ± 7.0	29.3 ± 9.0	35.0 ± 2.3	21.0 ± 7.5	15.0 ± 4.2	15.5 ± 4.0

## Data Availability

Data is contained within the article and Supplementary Materials.

## Supplementary Materials

The following supporting information can be downloaded at https://www.mdpi.com/article/10.3390/pharmaceutics16070927/s1, Table S1. Untargeted metabolic profiling of Lentisk oil by ESI(+) FT-ICR MS; Table S2. Untargeted metabolic profiling of Lentisk oil by ESI(-) FT-ICR MS; Table S3. Relative abundance of Lentisk seed oil components revealed in static-HS/GC-MS analysis; Table S4. Relative abundance of Lentisco seed oil components revealed in HS-SPME/GC-MS analysis; Figure S1. LVX release profile from NEsL by cellulose dialysis tubing.
