# Supplementary material for: Lentisk (Pistacia lentiscus) Oil Nanoemulsions Loaded with Levofloxacin: Phytochemical Profiles and Antibiofilm Activity against Staphylococcus spp
Source: Pharmaceutics. 2024 Jul 11;16(7):927. doi: 10.3390/pharmaceutics16070927 (PMC11280327; doi:10.3390/pharmaceutics16070927)
Supplement: Supplementary file 1 [file pharmaceutics-16-00927-s001.zip › pharmaceutics-3060004-supplementary.pdf]

Supplementary Materials:

Table S1: Untargeted metabolic profiling of Lentisk oil by ESI(+) FT-ICR MS

#	Putative Annotation	Ion	Molecular Formula	Theo. *m/z* [a]	Exp. *m/z* [b]	Neutral *m/z*	Intensity
1	Alanine	[M+H]+	C3H7NO2	90.05496	90.05609	89.04768	5.85E+04
2	Choline	M+	C5H14NO	104.10699	104.10699	104.10754	1.08E+05
3	Succinaldehyde	[M+Na]+	C4H6O2	109.02600	109.02642	86.03678	4.89E+04
4	Dimethyl-beta-alanine	[M+H]+	C5H10NO2	117.07843	117.08008	116.07115	6.88E+04
5	Succinic acid	[M+H]+	C4H6O4	119.03389	119.03417	118.02661	9.15E+04
6	Hydroxycadaverine	[M+H]+	C5H15N2O	120.12572	120.12577	119.11844	5.14E+04
7	Nicotinic acid	[M+H]+	C6H5NO2	124.03931	124.04009	123.03203	5.90E+04
8	Isovaleraldehyde	[M+K]+	C5H10O	125.03632	125.03684	86.07316	7.31E+04
9	Heptanoate	[M+H]+	C7H13O2	130.09938	130.09993	129.09210	5.51E+04
10	Diethylpyrazine	[M+H]+	C8H12N2	137.10733	137.10869	136.10005	7.54E+04
11	Monoterpene	[M+H]+	C10H16	137.13248	137.13247	136.12520	5.86E+04
12	S-oxocysteine	[M+H]+	C3H7NO3S	138.02194	138.02360	137.01466	6.05E+04
13	Nonenal	[M+H]+	C9H16O	141.12739	141.12899	140.12012	1.86E+05
14	Amino 2-oxopropanoate	[M+K]+	C3H5NO3	141.99010	141.98967	103.02694	4.18E+04
15	Hydroxypropanedioic acid	[M+Na]+	C3H4O5	142.99509	142.99449	120.00587	4.11E+04
16	Dimethyl fumarate	[M+H]+	C6H8O4	145.04953	145.04933	144.04226	5.80E+04
17	Butyl isobutyrate	[M+H]+	C8H16O2	145.12230	145.12205	144.11503	4.83E+04
18	Maltol	[M+Na]+	C6H6O3	149.02091	149.02020	126.03169	4.26E+04
19	Heptanoic acid	[M+Na]+	C7H14O2	153.08860	153.08824	130.09938	4.59E+04
20	Maleylacetate	[M+H]+	C6H6O5	159.02880	159.02978	158.02152	3.85E+04
21	Dihydroxyquinoline	[M+H]+	C9H7NO2	162.05496	162.05668	161.04768	4.23E+04
22	Pterin	[M+H]+	C6H5N5O	164.05669	164.05745	163.04941	3.81E+04
23	Conhydrinone	[M+Na]+	C8H15NO	164.10458	164.10434	141.11536	3.99E+04
24	Heptenoic acid	[M+K]+	C7H12O2	167.04689	167.04676	128.08373	7.17E+04
25	Limonene aldehyde	[M+H]+	C11H18O	167.14304	167.14363	166.13577	3.52E+04
26	Norspermidine	[M+K]+	C6H17N3	170.10541	170.10487	131.14225	4.40E+04
27	N-Dodecane	[M+H]+	C12H26	171.21073	171.21158	170.20345	9.35E+04
28	Capric acid	[M+H]+	C10H20O2	173.15361	173.15389	172.14633	3.39E+04
29	Cysteine sulfinic acid	[M+Na]+	C3H7NO4S	175.99880	175.99886	153.00958	2.95E+04
30	Ethyl maltol	[M+K]+	C7H8O3	179.01050	179.01060	140.04734	3.18E+04
31	Octanediol	[M+K]+	C8H18O2	185.09384	185.09426	146.13068	1.38E+05
32	Dimethyl-ethylazulene	[M+H]+	C14H16	185.13248	185.13169	184.12520	4.39E+04
33	S-Propyl-L-cysteine	[M+Na]+	C6H13NO2S	186.05592	186.05571	163.06670	2.74E+04
34	Thymol	[M+K]+	C10H14O	189.06762	189.06837	150.10447	5.57E+04
35	Undecenal	[M+Na]+	C11H20O	191.14064	191.13991	168.15142	2.82E+04
36	Pentyl-furanone	[M+K]+	C9H14O2	193.06254	193.06181	154.09938	3.06E+04
37	S-(Allylthio)-L-cysteine	[M+H]+	C6H11NO2S2	194.03040	194.03088	193.02312	3.42E+04
38	Glucuronic acid	[M+H]+	C6H10O7	195.04993	195.05138	194.04265	2.87E+04
39	Glycylproline	[M+Na]+	C7H12N2O3	195.07401	195.07369	172.08479	5.06E+04
40	Sulforaphene	[M+Na]+	C6H9NOS2	198.00178	198.00254	175.01256	5.08E+04
41	Cadalene	[M+H]+	C15H18	199.14813	199.14960	198.14085	3.25E+04
42	Boschniakine	[M+K]+	C10H11NO	200.04722	200.04731	161.08406	2.54E+04
43	p-coumaric acid	[M+K]+	C9H8O3	203.01050	203.01109	164.04735	3.39E+04
44	Serylproline	[M+H]+	C8H14N2O4	203.10263	203.10202	202.09536	4.39E+04
45	Sebacic acid	[M+H]+	C10H18O4	203.12779	203.12755	202.12051	2.48E+04
46	Phenyl-Alanine	[M+K]+	C9H11NO2	204.04214	204.04287	165.07898	3.02E+04
47	Homocysteic acid	[M+Na]+	C4H9NO5S	206.00936	206.01020	183.02014	3.17E+04
48	N-Acetyl-phenylalanine	[M+H]+	C11H13NO3	208.09682	208.09781	207.08954	2.53E+04
49	Flavan	[M+H]+	C15H14O	211.11174	211.11072	210.10447	3.24E+04
50	Hydroxycitrulline	[M+Na]+	C6H13N3O4	214.07983	214.07994	191.09061	2.70E+04
51	Hexyl cinnamaldehyde	[M+H]+	C15H20O	217.15869	217.15912	216.15142	2.79E+04
52	Hydroxydodecanoic acid	[M+H]+	C12H24O3	217.17982	217.18054	216.17254	2.80E+04
53	Caffeic acid	[M+K]+	C9H8O4	219.00542	219.00441	180.04226	2.74E+04
54	Hexose	[M+K]+	C6H12O6	219.02655	219.02621	180.06339	2.99E+04
55	Dihydroferulic acid	[M+Na]+	C10H12O4	219.06278	219.06296	196.07356	3.13E+04
56	Hydroxy-methylguanine	[M+K]+	C6H7N5O2	220.02313	220.02318	181.05597	1.35E+05
57	Caryophyllene alpha-oxide	[M+H]+	C15H24O	221.18999	221.18974	220.18272	2.82E+04

#	Putative Annotation	Ion	Molecular Formula	Theo. *m/z* [a]	Exp. *m/z* [b]	Neutral *m/z*	Intensity
**58**	Acetamido-5-hydroxy-2-(sulfanylmethyl)pentanoic acid	[M+H]+	C8H15NO4S	222.07945	222.08028	221.07218	2.45E+04
**59**	Flavanone	[M+H]+	C15H12O2	225.09100	225.09041	224.08373	3.12E+04
**60**	Allyl cinnamate	[M+K]+	C12H12O2	227.04689	227.04680	188.08373	2.68E+04
**61**	Hydroxy-trimethyl-lysine	[M+Na]+	C9H21N2O3	228.14444	228.14501	205.15522	2.93E+04
**62**	N-Decanoylglycine	[M+H]+	C12H23NO3	230.17507	230.17610	229.16779	2.98E+04
**63**	Valylhydroxyproline	[M+H]+	C10H18N2O4	231.13393	231.13486	230.12666	4.09E+04
**64**	Sampangine	[M+H]+	C15H8N2O	233.07094	233.07108	232.06366	7.31E+04
**65**	Marindinin	[M+H]+	C14H16O3	233.11722	233.11771	232.10994	5.77E+04
**66**	Cysteinyl-Isoleucine	[M+H]+	C9H18N2O3S	235.11109	235.11264	234.10381	6.92E+04
**67**	2-amino-3-(2-hydroxy-6-oxopyrimidin-1-yl)propanoic acid	[M+K]+	C7H9N3O4	238.02246	238.02156	199.05931	3.40E+04
**68**	Glycero-galacto-Octulose	[M+H]+	C8H16O8	241.09179	241.09272	240.08452	3.03E+04
**69**	Cytosine phosphate	[M+K]+	C4H2N3O5P	241.93637	241.93600	202.97321	3.71E+04
**70**	Indolepyruvate	[M+K]+	C11H9NO3	242.02140	242.02075	203.05824	3.87E+04
**71**	Dihydroxy-naphthoic acid	[M+K]+	C11H8O4	243.00542	243.00595	204.04226	4.51E+04
**72**	Aspartyl-phosphate	[M+K]+	C4H8NO7P	251.96700	251.96619	213.00384	2.77E+04
**73**	Glucose propionate	[M+H]+	C9H16O8	253.09179	253.09230	252.08452	2.87E+04
**74**	Djenkolic acid	[M+H]+	C7H14N2O4S2	255.04678	255.04825	254.03950	3.38E+04
**75**	Phomarin	[M+H]+	C15H10O4	255.06519	255.06751	254.05791	2.87E+04
**76**	Galactosyl glycerol	[M+H]+	C9H18O8	255.10745	255.10840	254.10017	3.88E+04
**77**	Hydroxydodecanoic acid	[M+K]+	C12H24O3	255.13570	255.13629	216.17254	3.53E+04
**78**	Oxalylalbizziine	[M+K]+	C6H9N3O6	258.01229	258.01299	219.04914	3.68E+04
**79**	Leucyl-Lysine	[M+H]+	C12H25N3O3	260.19687	260.19558	259.18959	4.03E+04
**80**	Mercaptolactate-cysteine disulfide	[M+Na]+	C6H11NO5S2	263.99708	263.99770	241.00786	3.39E+04
**81**	*p* -Coumaric acid sulfate	[M+Na]+	C9H8O6S	266.99338	266.99382	244.00416	2.86E+04
**82**	Dihydrocitrinone	[M+H]+	C13H14O6	267.08631	267.08751	266.07904	3.54E+04
**83**	Gingerol	[M+H]+	C15H22O4	267.15908	267.15868	266.15181	3.42E+04
**84**	Octadecenal	[M+H]+	C18H34O	267.26824	267.26698	266.26097	2.98E+04
**85**	Adenosine	[M+H]+	C10H13N5O4	268.10403	268.10552	267.09675	5.07E+04
**86**	Fructose lactate	[M+H]+	C9H16O9	269.08671	269.08753	268.07943	3.61E+04
**87**	Ellipticine	[M+Na]+	C17H14N2	269.10492	269.10497	246.11570	3.17E+04
**88**	Aspartyl-Asparagine	[M+Na]+	C8H13N3O6	270.06966	270.06986	247.08044	3.24E+04
**89**	Methionylvaline	[M+Na]+	C10H20N2O3S	271.10868	271.10774	248.11946	1.13E+05
**90**	Glutaminyllysine	[M+H]+	C11H22N4O4	275.17138	275.17233	274.16411	3.76E+04
**91**	N7-(2-Carbamoyl-2-hydroxyethyl)guanine	[M+K]+	C8H10N6O3	277.04460	277.04393	238.08144	3.11E+04
**92**	Lubiminol	[M+Na]+	C15H26O3	277.17741	277.17766	254.18819	3.39E+04
**93**	Decenoylcholine	[M+Na]+	C15H30NO2	279.21687	279.21765	256.22765	6.61E+04
**94**	Cysteinyl-Histidine	[M+Na]+	C9H14N4O3S	281.06788	281.06842	258.07866	4.02E+04
**95**	Octadecenoic acid	[M+H]+	C18H33O2	282.25588	282.25453	281.24860	3.02E+04
**96**	Hydroxyprolyl-Leucine	[M+K]+	C11H20N2O4	283.10547	283.10481	244.14231	3.23E+04
**97**	Lenticin	[M+K]+	C14H18N2O2	285.09999	285.10041	246.13683	3.34E+04
**98**	Alpha-Amylcinnamyl acetate	[M+K]+	C16H22O2	285.12514	285.12571	246.16198	4.45E+04
**99**	Lansiumamide B	[M+Na]+	C18H17NO	286.12023	286.12010	263.13101	4.05E+04
**100**	Piperine	[M+H]+	C17H19NO3	286.14377	286.14413	285.13649	5.13E+04
**101**	Octenoylcarnitine	[M+H]+	C15H27NO4	286.20129	286.20335	285.19401	3.34E+04
**102**	Hydroxyindoleacetylglycine	[M+K]+	C12H12N2O4	287.04286	287.04379	248.07916	4.37E+04
**103**	Vitamin A	[M+H]+	C20H30O	287.23694	287.23607	286.22967	3.07E+04
**104**	Lysyl-Phenylalanine	[M+H]+	C15H23N3O3	294.18122	294.18373	293.17394	3.12E+04
**105**	Methyl stearate	[M+H]+	C19H38O2	299.29446	299.29567	298.28718	4.39E+04
**106**	N-Caffeoyltyramine	[M+H]+	C17H17NO4	300.12303	300.12545	299.11576	5.26E+04
**107**	Pentadecanoylglycine	[M+H]+	C17H33NO3	300.25332	300.25318	299.24604	5.61E+04
**108**	Glyceryl 5-hydroxydecanoate	[M+K]+	C13H26O5	301.14118	301.14128	262.17802	4.36E+05
**109**	Gravolenic acid	[M+Na]+	C14H16O6	303.08391	303.08473	280.09469	3.40E+04
**110**	Linoleic acid	[M+Na]+	C18H32O2	303.22945	303.22919	280.24023	8.55E+04
**111**	Oleic acid	[M+Na]+	C18H34O2	305.24510	305.24474	282.25588	1.58E+05
**112**	Hexadecanedioic acid	[M+Na]+	C16H30O4	309.20363	309.20212	286.21441	6.25E+04
**113**	Lauroyl diethanolamide	[M+Na]+	C16H33NO3	310.23526	310.23439	287.24604	1.44E+05

#	Putative Annotation	Ion	Molecular Formula	Theo. *m/z* [a]	Exp. *m/z* [b]	Neutral *m/z*	Intensity
**114**	Fukiic acid	[M+K]+	C11H12O8	311.01638	311.01502	272.05322	4.32E+04
**115**	Arbutin	[M+K]+	C12H16O7	311.05276	311.05427	272.08961	1.42E+05
**116**	Dehydrosalvipisone	[M+H]+	C20H22O3	311.16417	311.16398	310.15690	3.97E+04
**117**	Valdiic acid	[M+H]+	C17H26O5	311.18530	311.18727	310.17802	3.99E+04
**118**	Nandigerine	[M+H]+	C18H17NO4	312.12303	312.12377	311.11576	6.89E+04
**119**	Pteroic acid	[M+H]+	C14H12N6O3	313.10436	313.10451	312.09709	4.09E+04
**120**	Hydroperoxylinoleic acid	[M+H]+	C18H32O4	313.23733	313.23633	312.23006	4.58E+04
**121**	Geranyl-diphosphate	[M+H]+	C10H20O7P2	315.07570	315.07614	314.06843	5.96E+04
**122**	Monatin	[M+Na]+	C14H16N2O5	315.09514	315.09595	292.10592	6.28E+04
**123**	N-Methylcarboxamidoadenosine	[M+Na]+	C11H14N6O4	317.09687	317.09807	294.10765	5.16E+04
**124**	Hydroxylinolenic acid	[M+Na]+	C18H30O3	317.20871	317.20866	294.21950	5.29E+04
**125**	N-Acetyldjenkolic acid	[M+Na]+	C9H16N2O5S2	319.03928	319.03931	296.05006	3.76E+04
**126**	Pentadecanoylglycine	[M+Na]+	C17H33NO3	322.23526	322.23667	299.24604	3.36E+04
**127**	Isoleucyl-prolyl-proline	[M+H]+	C16H27N3O4	326.20743	326.20712	325.20016	4.31E+04
**128**	Dihydroxyphenylvaleric acid 4 sulfate	[M+K]+	C11H14O7S	329.00918	329.00967	290.04602	5.19E+04
**129**	Dillanol	[M+Na]+	C18H12O5	331.05769	331.05666	308.06847	3.40E+04
**130**	Gingerdione	[M+K]+	C17H24O4	331.13062	331.13144	292.16746	4.20E+04
**131**	Sinapine	[M+Na]+	C16H24NO5	333.15467	333.15590	310.16545	3.45E+04
**132**	Isolinderenolide	[M+H]+	C21H34O3	335.25807	335.26053	334.25080	4.25E+04
**133**	1-methylguanosine	[M+K]+	C11H15N5O5	336.07048	336.07120	297.10732	4.63E+04
**134**	N-Myristoyl Serine	[M+Na]+	C17H33NO4	338.23018	338.23073	315.24096	7.85E+04
**135**	Glycyl-prolyl-glutamic acid	[M+K]+	C12H19N3O6	340.09054	340.09090	301.14739	5.10E+04
**136**	Deca-trienedioylcarnitine	[M+H]+	C17H25NO6	340.17546	340.17509	339.16819	4.22E+04
**137**	Asparaginyl-glycyl-aspartic acid	[M+K]+	C10H16N4O7	343.06506	343.06525	304.10190	3.47E+04
**138**	Matricarin	[M+K]+	C17H20O5	343.09423	343.09410	304.13107	5.97E+04
**139**	Sucrose	[M+H]+	C12H22O11	343.12349	343.12565	342.11621	3.95E+04
**140**	Rosmaricine	[M+H]+	C20H27NO4	346.20128	346.20008	345.19401	3.84E+04
**141**	Gibberellic acid	[M+H]+	C19H22O6	347.14891	347.14826	346.14164	7.51E+04
**142**	Dimethylguanosine	[M+K]+	C12H17N5O5	350.08613	350.08599	311.12297	4.32E+04
**143**	Trihydroxystearic acid	[M+Na]+	C18H36O5	355.24549	355.24625	332.25627	3.52E+04
**144**	Pipericine	[M+Na]+	C22H41NO	358.30803	358.30720	335.31881	6.49E+04
**145**	Curcumin III	[M+Na]+	C21H20O4	359.12538	359.12463	336.13616	4.95E+04
**146**	Epoxyeicosa-enoic acid	[M+K]+	C20H36O3	363.22960	363.22854	324.26645	4.00E+04
**147**	Docosanal	[M+K]+	C22H44O	363.30237	363.30285	324.33922	3.93E+04
**148**	4'-Nitrophenyl-2-acetamido-2-deoxy-beta-glucopyranoside	[M+Na]+	C14H18N2O8	365.09554	365.09458	342.10632	1.66E+05
**149**	Methyl 6-O-galloyl-beta-D-glucopyranoside	[M+Na]+	C14H18O10	369.07922	369.07769	346.09000	1.13E+05
**150**	Mytilin A	[M+K]+	C13H20N2O8	371.08512	371.08497	332.12197	6.85E+04
**151**	Gibberellin A53	[M+Na]+	C20H28O5	371.18289	371.18302	348.19367	4.71E+04
**152**	Glycerylmonooleate	[M+H]+	C21H38O5	371.27920	371.28004	370.27192	3.72E+04
**153**	Dihydroferulic acid 4-O-glucuronide	[M+H]+	C16H20O10	373.11292	373.11445	372.10565	9.23E+04
**154**	Tsangane L 3-glucoside	[M+H]+	C19H34O7	375.23773	375.24065	374.23045	4.11E+04
**155**	Bergamottin	[M+K]+	C21H22O4	377.11497	377.11446	338.15181	3.93E+04
**156**	Resmethrin	[M+K]+	C22H26O3	377.15135	377.15113	338.18820	4.30E+04
**157**	Hydroxygaleon	[M+K]+	C20H22O5	381.10988	381.11106	342.14672	3.94E+04
**158**	Naringenin sulfate	[M+K]+	C15H12O8S	390.98845	390.98788	352.02529	7.27E+04
**159**	Moschamine	[M+K]+	C20H20N2O4	391.10547	391.10526	352.14231	1.02E+05
**160**	Gingerdiol diacetate	[M+K]+	C19H28O6	391.15175	391.15328	352.18859	7.91E+04
**161**	Tricosanedione	[M+K]+	C23H44O2	391.29729	391.29573	352.33413	9.08E+04
**162**	Chlorogenic acid	[M+K]+	C16H18O9	393.05824	393.05932	354.09508	2.99E+05
**163**	Salicyluric beta-D-glucuronide	[M+Na]+	C15H17NO10	394.07447	394.07603	371.08525	6.64E+04
**164**	Tetrahydropalmatine	[M+K]+	C21H25NO4	394.14152	394.14197	355.17836	4.79E+04
**165**	Dihydroxydodecanoylcarnitine	[M+Na]+	C19H37NO6	398.25131	398.25240	375.26209	4.42E+04
**166**	Linoleoyl Proline	[M+Na]+	C23H39NO3	400.28221	400.28090	377.29299	5.34E+04
**167**	Albafuran A	[M+Na]+	C24H26O4	401.17233	401.17251	378.18311	1.40E+05
**168**	Bufotenine O-glucoside	[M+K]+	C18H26N2O6	405.14224	405.14174	366.17909	8.68E+04
**169**	Pentadecenoylcarnitine	[M+Na]+	C22H41NO4	406.29278	406.29138	383.30356	3.67E+04
**170**	Feruloylquinic acid	[M+K]+	C17H20O9	407.07389	407.07228	368.11073	3.57E+04

#	Putative Annotation	Ion	Molecular Formula	Theo. *m/z* [a]	Exp. *m/z* [b]	Neutral *m/z*	Intensity
**171**	MG(0:0/20:1/0:0)	[M+Na]+	C23H44O4	407.31318	407.31244	384.32396	9.14E+04
**172**	Apo-beta-carotenal	[M+K]+	C27H36O	415.23977	415.23969	376.27662	3.77E+04
**173**	Tylophorine	[M+Na]+	C24H27NO4	416.18323	416.18420	393.19401	6.39E+04
**174**	Naringenin rhamnoside	[M+H]+	C21H22O9	419.13366	419.13547	418.12638	4.33E+04
**175**	Di-4-coumaroylputrescine	[M+K]+	C22H24N2O4	419.13677	419.13547	380.17361	4.33E+04
**176**	Methyl 3,4-dihydroxy-5-prenylbenzoate 3-glucoside	[M+Na]+	C19H26O9	421.14690	421.14568	398.15768	4.71E+04
**177**	Docosahexaenoyl Alanine	[M+Na]+	C25H37NO3	422.26656	422.26625	399.27734	3.78E+04
**179**	3,4-Dimethyl-5-pentyl-2-furanpentadecanoic acid	[M+Na]+	C26H46O3	429.33392	429.33514	406.34470	3.89E+04
**180**	Solasodiene	[M+K]+	C27H41NO	434.28197	434.28255	395.31882	4.34E+04
**181**	Schisandrin B	[M+K]+	C23H28O6	439.15175	439.15219	400.18859	4.08E+04
**182**	Camellenodiol	[M+H]+	C29H46O3	443.35197	443.35110	442.34470	5.11E+04
**183**	Fusaproliferin	[M+H]+	C27H40O5	445.29485	445.29468	444.28757	5.54E+04
**184**	Hydroxy-gamma-tocotrienol	[M+Na]+	C28H42O3	449.30262	449.30234	426.31340	3.55E+04
**185**	Hydroxycalcitriol	[M+Na]+	C27H44O4	455.31318	455.31338	432.32396	3.61E+04
**186**	Kaempferol 3-O-arabinoside	[M+K]+	C20H18O10	457.05315	457.05307	418.09000	4.85E+04
**187**	Acetolein	[M+Na]+	C25H44O6	463.30301	463.30233	440.31379	6.72E+04
**188**	Artoflavanone	[M+K]+	C24H28O7	467.14666	467.14617	428.18350	5.60E+04
**189**	Austalide J	[M+Na]+	C25H32O7	467.20402	467.20361	444.21480	5.47E+04
**190**	Vitamin K	[M+Na]+	C31H46O2	473.33900	473.33962	450.34978	4.40E+04
**191**	PA(10:0/8:0)	[M+Na]+	C21H41O8P	475.24313	475.24234	452.25391	1.03E+05
**192**	Glucoraphanin	[M+K]+	C12H23NO10S3	476.01157	476.01339	437.04840	6.05E+04
**193**	Fuziline	[M+Na]+	C24H39NO7	476.26187	476.26321	453.27265	5.58E+04
**194**	N-Docosahexaenoyl phenylalanine	[M+H]+	C31H41NO3	476.31592	476.31586	475.30864	5.05E+04
**195**	Alpha-Guttiferin	[M+Na]+	C27H34O6	477.22476	477.22393	454.23554	8.41E+04
**196**	O-Desmethylvenlafaxine glucuronide	[M+K]+	C22H33NO8	478.18378	478.18499	439.22062	4.26E+04
**197**	Pfaffic acid	[M+K]+	C29H44O3	479.29220	479.29409	440.32905	9.74E+04
**198**	Folic acid	[M+K]+	C19H19N7O6	480.10284	480.10358	441.13968	3.88E+04
**199**	Lythramine	[M+H]+	C29H37NO5	480.27445	480.27445	479.26717	4.78E+04
**200**	a-L-Fucopyranosyl-(1->2)-b-D-galactopyranosyl-(1->2)-D-xylose	[M+Na]+	C17H30O14	481.15278	481.15302	458.16356	5.83E+04
**201**	1-alpha,24R,25-Trihydroxyvitamin D2	[M+K]+	C28H44O4	483.28712	483.28565	444.32396	4.67E+04
**202**	LysoPG(16:0/0:0)	[M+H]+	C22H45O9P	485.28740	485.28921	484.28012	3.56E+04
**203**	Trimethylscutellarein 7-glucoside	[M+H]+	C24H26O11	491.15479	491.15722	490.14751	4.07E+04
**204**	Cymorcin diglucoside	[M+H]+	C22H34O12	491.22013	491.21448	490.20503	3.83E+04
**205**	Hexadecyl octadecenal	[M+H]+	C34H66O	491.51864	491.51808	490.51137	5.37E+04
**206**	Malvidin 3-glucoside	[M+H]+	C23H25O12	494.14188	494.14138	493.13460	4.51E+04
**207**	Alpha-L-Rhamnopyranosyl-(1->3)-alpha-D-galactopyranosyl-(1->3)-L-fucose	[M+Na]+	C18H32O14	495.16843	495.16756	472.17921	3.99E+04
**208**	Macrocarpal	[M+Na]+	C28H40O6	495.27171	495.26938	472.28249	3.81E+04
**209**	Carpaine	[M+Na]+	C28H50N2O4	501.36628	501.36558	478.37706	3.49E+04
**210**	Caryatin glucoside	[M+H]+	C24H26O12	507.14970	507.15002	506.14243	3.49E+04
**211**	LysoPG(18:2/0:0)	[M+H]+	C24H45O9P	509.28740	509.29001	508.28012	7.26E+04
**212**	Enterodiol glucuronide	[M+K]+	C24H30O10	517.14706	517.14921	478.18390	4.13E+04
**213**	Deterrol stearate	[M+K]+	C33H50O2	517.34424	517.34262	478.38108	4.15E+04
**214**	Dukunolide A	[M+K]+	C26H26O9	521.12084	521.12107	482.15768	7.64E+04
**215**	7,8-Dihydro-3b,6a-dihydroxy-alpha-ionol 9-[apiosyl-(1->6)-glucoside]	[M+H]+	C24H42O12	523.27490	523.27643	522.26763	5.72E+04
**216**	Garciduol	[M+K]+	C27H18O9	525.05824	525.05712	486.09508	5.04E+04
**217**	Methyl 1-(3,4-dimethoxyphenyl)-3-(3-ethylpentyl)-4-hydroxy-6,7,8-trimethoxynaphthalene-2-carboxylate	[M+H]+	C30H38O8	527.26395	527.26590	526.25667	4.05E+04
**218**	Pyrohyperforin	[M+H]+	C35H50O4	535.37819	535.37819	534.37091	7.57E+04
**219**	LysoPE(0:0/22:1)	[M+H]+	C27H54NO7P	536.37107	536.37177	535.36379	5.59E+04
**220**	LysoPC(18:3/0:0)	[M+Na]+	C26H48NO7P	540.30606	540.30778	517.31684	5.54E+04
**221**	LysoPE(0:0/20:1)	[M+K]+	C25H50NO7P	546.29565	546.29586	507.33249	4.33E+04
**222**	DG(15:0/0:0/16:1)	[M+H]+	C34H64O5	553.48265	553.48303	552.47375	4.00E+04

#	Putative Annotation	Ion	Molecular Formula	Theo. *m/z* [a]	Exp. *m/z* [b]	Neutral *m/z*	Intensity
**223**	Viniferal	[M+H]+	C35H26O8	575.17004	575.17194	574.16277	3.83E+04
**224**	D-Phenylalanyl-L-2-piperidinecarbonyl-N-(4-nitro phenyl)-L-argininamide	[M+Na]+	C27H36N8O5	575.27009	575.26830	552.28087	4.06E+04
**225**	Myricetin 3-(3'',4''-diacetylrhamnoside)	[M+K]+	C25H24O14	587.07976	587.07831	548.11661	4.74E+04
**226**	DG(14:0/0:0/20:5)	[M+H]+	C37H62O5	587.46700	587.46910	586.45973	3.94E+04
**227**	Cer(d18:1/18:0)	[M+Na]+	C36H71NO3	588.53262	588.53040	565.54340	4.62E+04
**228**	Quercetin 3-(6''-malonyl-glucoside)	[M+K]+	C24H22O15	589.05903	589.05873	550.09587	4.47E+04
**229**	Licuroside	[M+K]+	C26H30O13	589.13180	589.13215	550.16864	6.71E+04
**230**	N-benzyl-2-{[4-hydroxy-1-(1-methylindole-3-carbonyl)pyrrolidin-2-yl]formamido}-N-methyl-3-(naphthalen-2-yl)propanamide	[M+H]+	C36H36N4O4	589.28093	589.28214	588.27366	4.38E+04
**231**	Myricatin	[M+K]+	C22H16O15S	590.98415	590.98399	552.02099	6.48E+04
**232**	Ganoderic acid H	[M+Na]+	C32H44O9	595.28775	595.28721	572.29853	4.86E+04
**233**	Nigramide G	[M+K]+	C33H38N2O6	597.23614	597.23641	558.27299	5.11E+04
**234**	Ursolic acid (cis-3-O-hydroxycinnamoyl-)	[M+H]+	C39H54O5	603.40440	603.40347	602.39713	7.89E+04
**235**	LysoPC(24:1)	[M+H]+	C32H64NO7P	606.44931	606.45113	605.44204	7.88E+04
**236**	Quercetin 3-O-xylosyl-glucuronide	[M+H]+	C26H26O17	611.12427	611.12131	610.11700	4.75E+04
**237**	Dieporeticenin	[M+K]+	C37H64O4	611.44362	611.44325	572.48046	4.38E+04
**238**	Solasodine 3-O-beta-D-glucopyranoside	[M+K]+	C33H52NO7	613.33699	613.33768	574.37438	5.45E+04
**239**	Asterinic acid	[M+Na]+	C40H50O4	617.36013	617.36085	594.37091	1.01E+05
**240**	3-Caffeoylpelargonidin 5-glucoside	[M+Na]+	C30H27O13	618.13439	618.13415	595.14517	4.96E+04
**241**	Bullatacinone	[M+H]+	C37H66O7	623.48813	623.48888	622.48085	4.19E+04
**242**	Cer(d16:1/23:0)	[M+Na]+	C39H77NO3	630.57957	630.57776	607.59035	5.16E+04
**243**	DG(14:0/0:0/20:0)	[M+K]+	C37H72O5	635.50113	635.50008	596.53798	4.17E+04
**244**	23-O-beta-D-Glucopyranosyl-25-methyldolichosterone	M+	C35H58O10	638.40245	638.40368	638.40300	7.55E+04
**245**	Dauricine	[M+K]+	C38H44N2O6	663.28310	663.28191	624.31994	7.15E+04
**246**	Coroloside	[M+H]+	C35H54O12	667.36880	667.37069	666.36153	4.85E+04
**247**	Dipalmitoyl-s-glyceryl cysteine	[M+H]+	C38H73NO6S	672.52314	672.52147	671.51586	4.45E+04
**248**	Ginsenoside Rh5	[M+Na]+	C37H64O9	675.44425	675.44330	652.45503	6.02E+04
**249**	Leucodelphinidin 3-[galactosyl-(1->4)-glucoside]	[M+K]+	C27H34O18	685.13767	685.13744	646.17451	6.50E+04
**250**	Tejedine	[M+Na]+	C38H40N2O9	691.26260	691.26395	668.27338	5.78E+04
**251**	Skyrin xyloside	[M+Na]+	C35H26O14	693.12148	693.12187	670.13226	7.02E+04
**252**	Licorice glycoside E	[M+H]+	C35H35NO14	694.21303	694.21609	693.20575	4.49E+04
**253**	Dipalmitoyl-s-glyceryl cysteine	[M+Na]+	C38H73NO6S	694.50508	694.50380	671.51586	4.99E+04
**254**	TG(10:0/22:0/8:0)	[M+H]+	C43H82O6	695.61842	695.61873	694.61114	4.09E+04
**255**	GlcCer(d14:2/20:0)	[M+H]+	C40H75NO8	698.55654	698.55980	697.54927	4.64E+04
**256**	Glycerol 1-(9Z-octadecenoate) 2-octanoate 3-tetradecanoate	[M+Na]+	C43H80O6	715.58471	715.58603	692.59549	6.57E+04
**257**	Glucosyl passiflorate	[M+Na]+	C37H60O12	719.39770	719.39551	696.40848	4.66E+04
**258**	Lecithin	[M+Na]+	C38H74NO8P	726.50442	726.50178	703.51521	6.72E+04
**259**	Glabrin D	[M+K]+	C36H49N7O10	778.31725	778.31552	739.35409	6.91E+04
**260**	Delphinidin 3-sophoroside 5-glucoside	[M+H]+	C33H41O22	790.21622	790.21691	789.20895	1.08E+05
**261**	PA(18:3/22:1)	[M+K]+	C43H77O8P	791.49876	791.49757	752.53561	6.47E+04
**262**	3-Digalloyl-4,5-di-O-galloylquinic acid	[M+H]+	C35H28O22	801.11450	801.11268	800.10722	5.06E+04
**263**	Kaempferol 3-(2''-rhamnosyl-6''-acetylgalactoside) 7-rhamnoside	[M+K]+	C35H42O20	821.19010	821.18803	782.22694	6.15E+04
**264**	2-methoxy-6-all trans-decaprenyl-2-methoxy-1,4-benzoquinol	[M+H]+	C57H88O3	821.68062	821.68463	820.67335	6.94E+04
**265**	Lucyoside J	[M+K]+	C42H66O15	849.40333	849.40304	810.44017	9.05E+04
**266**	Lyciumin A	[M+H]+	C42H51N9O12	874.37299	874.37392	873.36572	5.81E+05
**267**	Lucyoside M	[M+K]+	C44H68O15	875.41898	875.41716	836.45582	2.02E+05
**268**	Isofucosterol 3-O-[6-O-(9,12-Octadecadienoyl)-b-D-glucopyranoside]	[M+K]+	C53H88O7	875.61616	875.61511	836.65301	1.18E+05
**269**	TG(18:3/18:4/18:3)	[M+Na]+	C57H90O6	893.66296	893.65903	870.67374	6.04E+04
**270**	3-hydroxyheptanoyl-CoA	[M+H]+	C28H48N7O18P3S	896.20621	896.20783	895.19894	1.73E+05

#	Putative Annotation	Ion	Molecular Formula	Theo. *m/z* [a]	Exp. *m/z* [b]	Neutral *m/z*	Intensity
**271**	N-(3,4-dihydroxy-1-{[3,4,5-trihydroxy-6-(hydroxymethyl)oxan-2-yl]oxy}octadecan-2-yl)hexacosanamide	[M+K]+	C50H99NO9	896.69514	896.69512	857.73198	6.50E+04
**272**	Trigonelloside C	[M+Na]+	C57H98O6	901.72556	901.72742	878.736341	1.63E+05
**273**	Linolein	[M+Na]+	C57H98O6	901.72556	901.72742	857.731984	1.63E+05
**274**	Catechin-(4alpha->8)-gallocatechin-(4alpha->8)-gallocatechin	[M+Na]+	C45H38O20	921.18486	921.18556	898.195644	1.17E+05
**275**	TG(18:4/22:2/18:0)	[M+H]+	C61H108O5	921.82695	921.82502	920.819677	7.15E+04
**276**	Lyciumin B	[M+K]+	C44H52N10O11	935.34486	935.34329	896.381703	7.26E+04
**277**	Theaflavonin	[M+H]+	C43H36O24	937.16693	937.16669	936.159652	1.05E+05
**278**	Hoduloside VIII	[M+Na]+	C46H76O18	939.49239	939.49133	916.503166	8.53E+04
**279**	Quercetin 3-coumaroyl-triglucoside	[M+H]+	C42H48O25	953.25574	953.25421	952.248467	6.09E+04
**280**	Betavulgaroside VIII	[M+Na]+	C46H68O20	963.41961	963.42013	940.430395	5.44E+04
**281**	TG(22:0/21:0/18:0)	[M+Na]+	C64H124O6	1011.92901	1011.93385	988.939792	4.93E+04
**282**	TG(20:3/22:5/22:1)	[M+H]+	C67H112O6	1013.85317	1013.85473	1012.84589	2.22E+05
**283**	Fevicordin B 2-[rhamnosyl-(1->4)-glucosyl-(1->6)-glucoside]	[M+H]+	C49H74O22	1015.47445	1015.47535	1014.46717	1.13E+05
**284**	Chrysoeriol 7-[feruloyl-(->2)-glucuronyl-(1->2)-glucuronyl-(1->3)-glucuronide]	[M+Na]+	C44H44O27	1027.19622	1027.19779	1004.207	4.86E+04
**285**	S-(2,3-Bis(palmitoyloxy)propyl)-N-palmitoylcysteinyl-alanyl-glycine	[M+Na]+	C59H111N3O9S	1060.79332	1060.79425	1037.8041	4.55E+04
**286**	Camelliatannin G	[M+H]+	C49H34O29	1087.12585	1087.12983	1086.11858	7.07E+04
**287**	TG(22:2/24:1/22:6)	[M+Na]+	C71H120O6	1091.89771	1091.89579	1068.90849	1.22E+05

[a]Theoretical m/z

[b]Experimental m/z

CerCeramide; DG Diacylglycerol; GalCer Galactosylceramide; GDP guanine diphosphate; MG Monoacylglycerol; PA Phosphatidic acid; PC Phosphatidylcholine; PG Glycerophospholipids; PI Phosphatidylinositol; PS Phosphatidylserine; TG Triacylglycerol.

Table S2: Untargeted metabolic profiling of Lentisk oil by ESI(-) FT-ICR MS

#	Putative Annotation	Ion	Molecular Formula	Theo. *m/z* [a]	Exp. *m/z* [b]	Neutral *m/z*	Intensity
1	Glyceric acid	[M-H]-	C3H6O4	105.01933	105.02001	106.02661	5.75E+04
2	Allyl methyl sulfide	[M+Cl]-	C4H8S	123.00407	123.00492	88.03467	4.64E+04
3	Methylcatechol	[M-H]-	C7H8O2	123.04516	123.04555	124.05243	4.73E+04
4	Isopentanol	[M+Cl]-	C5H12O	123.05822	123.05884	88.08882	5.60E+04
5	Heptenoic acid	[M-H]-	C7H12O2	127.07645	127.07773	128.08373	9.93E+04
6	(2-amino-2-methylpropyl)(isopropyl)amine	[M-H]-	C7H18N2	129.13972	129.14008	130.14700	5.11E+04
7	N-Acetyl-L-alanine	[M-H]-	C5H9NO3	130.05097	130.05113	131.05824	1.45E+05
8	Bran oil	[M+Cl]-	C5H4O2	130.99053	130.99077	96.02113	4.43E+04
9	Deoxyguanine	[M-H]-	C5H7N5	136.06287	136.06213	137.07015	3.35E+04
10	Dimethylglycine	[M+Cl]-	C4H9NO2	138.03273	138.03402	103.06333	4.50E+04
11	2-Mercaptopropanoic acid	[M+Cl]-	C3H6O2S	140.97825	140.97803	106.00885	3.76E+04
12	Hydroxymaltol	[M-H]-	C6H6O4	141.01933	141.01990	142.02661	5.92E+04
13	S-Nitrosomercaptoethanol	[M+Cl]-	C2H5NO2S	141.97350	141.97394	107.00410	5.19E+04
14	Nonanol	[M-H]-	C9H20O	143.14414	143.14508	144.15142	6.22E+04
15	Catechol	[M+Cl]-	C6H6O2	145.00618	145.00702	110.03678	3.58E+04
16	Oil garlic	[M+Cl]-	C6H10S	149.01972	149.02088	114.05032	5.23E+04
17	Carvacrol	[M-H]-	C10H14O	149.09719	149.09815	150.10447	3.75E+04
18	Cinnamaldehyde	[M+Cl]-	C9H9	152.03983	152.04058	117.07043	4.46E+04
19	S-1-Propenyl thiosulfate	[M-H]-	C3H6O3S2	152.96856	152.96864	153.97584	4.40E+04
20	3-Phenylpyridine	[M-H]-	C11H9N	154.06622	154.06695	155.07350	4.30E+04
21	Purine	[M+Cl]-	C5H4N4	155.01300	155.01413	120.04360	1.24E+05
22	2-amino-2,3-dihydroxypropanoic acid	[M+Cl]-	C3H7NO4	156.00691	156.00742	121.03751	3.50E+04
23	Dimethylmalate	[M-H]-	C6H8O5	159.03100	159.03193	160.03827	9.66E+04
24	Taurine	[M+Cl]-	C2H7NO3S	159.98407	159.98331	125.01466	3.82E+04
25	Nicotine imine	[M-H]-	C10H13N2	160.10060	160.10015	161.10787	3.45E+04
26	1-Methylpropyl 1-propenyl disulfide	[M-H]-	C7H14S2	161.04642	161.04668	162.05369	6.02E+04
27	Rhamnose	[M-H]-	C6H12O5	163.06120	163.06065	164.06847	2.52E+04
28	Vanillic acid	[M-H]-	C8H8O4	167.03498	167.03338	168.04226	1.17E+05
29	Citronellic acid	[M-H]-	C10H18O2	169.12340	169.12508	170.13068	2.08E+04
30	amino 2-amino-3-sulfanylpropanoate	[M+Cl]-	C3H8N2O2S	171.00005	170.99901	136.03065	8.16E+04
31	1,2-Dihydro-1,1,6-trimethylnaphthalene	[M-H]-	C13H16	171.11793	171.11748	172.12520	3.16E+04
32	Salicylates	[M+Cl]-	C7H5O3	171.99382	171.99471	137.02442	4.06E+04
33	Brassilexin	[M-H]-	C9H6N2S	173.01789	173.01875	174.02517	3.25E+04
34	Hexanethioic acid S-propyl ester	[M-H]-	C9H18OS	173.10056	173.10089	174.10784	3.85E+04
35	Hypoglycin A	[M+Cl]-	C7H11NO2	176.04838	176.04947	141.07898	2.53E+04
36	7-Aminomethyl-7-carbaguanine	[M-H]-	C7H9N5O	178.07343	178.07407	179.08071	3.49E+04
37	Hexose	[M-H]-	C6H12O6	179.05611	179.05636	180.06339	2.91E+04
38	Garlicin	[M+Cl]-	C6H10S2	180.99179	180.99222	146.02239	5.16E+04
39	Glutamic acid	[M+Cl]-	C5H9NO4	182.02256	182.02316	147.05316	2.42E+04
40	(S)-Actinidine	[M+Cl]-	C10H13N	182.07420	182.07539	147.10480	1.97E+04
41	Nornicotine	[M+Cl]-	C9H12N2	183.06945	183.06900	148.10005	2.56E+04
42	Acetylspermidine	[M-H]-	C9H21N3O	186.16119	186.16237	187.16846	1.86E+04
43	Gentisic acid	[M+Cl]-	C7H6O4	188.99601	188.99661	154.02661	3.26E+04
44	2,5-Furandicarboxylic acid	[M+Cl]-	C6H4O5	190.97528	190.97367	156.00587	4.44E+04
45	Nonenoic acid	[M+Cl]-	C9H16O2	191.08443	191.08601	156.11503	6.03E+04
46	S-Butylcysteine sulfoxide	[M-H]-	C7H15NO3S	192.06999	192.07164	193.07726	2.70E+04
47	Monopropionylcadaverine	[M+Cl]-	C8H18N2O	193.11132	193.11039	158.14191	3.32E+04
48	Hydroxystilbene	[M-H]-	C14H12O	195.08154	195.08234	196.08882	3.59E+04
49	S-(2-Aminoethyl)-L-cysteine	[M+Cl]-	C5H12N2O2S	199.03135	199.03181	164.06195	1.10E+05
50	Camalexin	[M-H]-	C11H8N2S	199.03354	199.03181	200.04082	1.10E+05
51	Phenylalanine	[M+Cl]-	C9H11NO2	200.04838	200.04829	165.07898	3.26E+04
52	Ethylphenyl sulfate	[M-H]-	C8H10O4S	201.02270	201.02353	202.02998	3.49E+04
53	Homocysteinesulfinic acid	[M+Cl]-	C4H9NO4S	201.99463	201.99354	167.02523	2.16E+04
54	N-Acetyl-S-allylcysteine	[M-H]-	C8H13NO3S	202.05434	202.05288	203.06161	1.86E+04
55	p-Menthane-tetrol glucoside	[M-H]-	C10H20O4	203.12888	203.12866	204.13616	2.43E+04
56	Phenyl ethyl-3-methyl butanote	[M-H]-	C13H18O2	205.12341	205.12450	206.13068	3.52E+04
57	Quinaldic acid	[M+Cl]-	C10H7NO2	208.01708	208.01694	173.04768	1.64E+04

#	Putative Annotation	Ion	Molecular Formula	Theo. *m/z* [a]	Exp. *m/z* [b]	Neutral *m/z*	Intensity
**58**	Glycero-galacto-heptitol	[M-H]-	C7H16O7	211.08233	211.08069	212.08960	2.08E+04
**59**	Methyleugenol	[M+Cl]-	C11H14O2	213.06878	213.07045	178.09938	2.76E+04
**60**	N-alpha-Acetyl-L-citrulline	[M-H]-	C8H15N3O4	216.09898	216.10099	217.10626	2.75E+04
**61**	1,2-O-Isopropylidene-D-glucofuranose	[M-H]-	C9H16O6	219.08741	219.08592	220.09469	2.85E+04
**62**	5-Methyl-2-phenyl-2-hexenal	[M+Cl]-	C13H16O	223.08952	223.09043	188.12012	1.85E+04
**63**	Linalyl butyrate	[M-H]-	C14H24O2	223.17036	223.16905	224.17763	1.75E+04
**64**	Resorcinol sulfate	[M+Cl]-	C6H6O5S	224.96300	224.96187	189.99359	2.60E+04
**65**	Alanylcysteine	[M+Cl]-	C6H12N2O3S	227.02627	227.02513	192.05686	2.05E+04
**66**	Myristic acid	[M-H]-	C14H28O2	227.20166	227.20188	228.20893	3.58E+04
**67**	Asparaginyl-Proline	[M-H]-	C9H15N3O4	228.09898	228.09828	229.10626	2.28E+04
**68**	Glucuronic acid	[M+Cl]-	C6H10O7	229.01206	229.01176	194.04265	2.99E+04
**69**	Gramineal	[M+Cl]-	C12H14N2O	237.08002	237.08104	202.11061	1.91E+04
**70**	Falcarinone	[M-H]-	C17H22O	241.15979	241.15769	242.16707	2.50E+04
**71**	Atractyligenin (2-O-beta-glucopyranosyl-)	[M-H]-	C12H21NO4	242.13978	242.13966	243.14706	3.26E+04
**72**	Isoleucyl-Isoleucine	[M-H]-	C12H24N2O3	243.17142	243.17361	244.17869	2.93E+04
**73**	Ellipticine	[M-H]-	C17H14N2	245.10842	245.10944	246.11570	3.62E+04
**74**	Cycloate	[M+Cl]-	C11H21NOS	250.10379	250.10177	215.13438	2.60E+04
**75**	2-Amino-N-(2,2,4,4-tetramethyl-3-thietanyl)propanamide	[M+Cl]-	C10H20N2OS	251.09904	251.09819	216.12963	3.20E+04
**76**	Cyperotundone	[M+Cl]-	C16H24	251.15720	251.15648	216.18780	2.82E+04
**77**	Propionylcarnitine	[M+Cl]-	C10H19NO4	252.10081	252.09832	217.13141	2.54E+04
**78**	Palmitoleic acid	[M-H]-	C16H30O2	253.21730	253.21685	254.22458	1.57E+04
**79**	N-Acetylalliin	[M+Cl]-	C8H13NO4S	254.02593	254.02462	219.05653	2.06E+04
**80**	Nicotinamide riboside	[M-H]-	C11H15N2O5	254.09082	254.08833	255.09810	2.03E+04
**81**	Purpurin	[M-H]-	C14H8O5	255.02990	255.02990	256.03717	4.03E+04
**82**	Palmitic acid	[M-H]-	C16H32O2	255.23296	255.23269	256.24023	3.13E+05
**83**	N-Acetyl-L-tyrosine	[M+Cl]-	C11H13NO4	258.05386	258.05133	223.08446	2.19E+04
**84**	2,4-Decadienoic isobutylamide	[M+Cl]-	C14H25NO	258.16302	258.16281	223.19361	1.81E+04
**85**	Dodecylglycerol	[M-H]-	C15H32O3	259.22787	259.22809	260.23514	2.10E+04
**86**	Vanilloylglycine	[M+Cl]-	C10H11NO5	260.03313	260.03524	225.06372	2.37E+04
**87**	Hydroxyprolyl-Methionine	[M-H]-	C10H18N2O4S	261.09145	261.09005	262.09873	2.20E+04
**88**	Glycyl-prolyl-glycinamide	[M+Cl]-	C9H16N4O3	263.09164	263.09265	228.12224	2.34E+04
**89**	Acetoxyeugenol acetate	[M-H]-	C14H16O5	263.09250	263.09265	264.09977	2.34E+04
**90**	1-(m-Methoxycinnamoyl)pyrrolidine	[M+Cl]-	C14H17NO2	266.09533	266.09614	231.12593	2.14E+04
**91**	Alantolactone	[M+Cl]-	C15H20O2	267.11573	267.11617	232.14633	2.19E+04
**92**	2-Propenyl 1-(2-propenylsulfinyl)propyl disulfide	[M+Cl]-	C9H16OS3	271.00573	271.00841	236.03633	2.58E+04
**93**	Hydroxyhexadecanoic acid	[M-H]-	C16H32O3	271.22787	271.22687	272.23514	4.46E+04
**94**	Glutaconylcarnitine	[M-H]-	C12H19NO6	272.11396	272.11528	273.12124	2.76E+04
**95**	2-(4-Hydroxyphenylazo)benzoic acid	[M+Cl]-	C13H10N2O3	277.03855	277.03950	242.06914	2.00E+04
**96**	Solanolone	[M-H]-	C15H18O5	277.10815	277.10923	278.11542	2.19E+04
**97**	Linolenic acid	[M-H]-	C18H30O2	277.21730	277.21693	272.22458	3.25E+04
**98**	Linoleic acid	[M-H]-	C18H32O2	279.23296	279.23047	280.24023	1.52E+05
**99**	Albendazole S-oxide	[M-H]-	C12H15N3O3S	280.07614	280.07675	281.08341	2.78E+04
**100**	Oleamide	[M-H]-	C18H35NO	280.26459	280.26329	281.27186	2.91E+04
**101**	Oleic acid	[M-H]-	C18H34O2	281.24861	281.24872	282.25588	7.10E+05
**102**	Stearic acid	[M-H]-	C18H36O2	283.26425	283.26432	284.227153	1.43E+05
**103**	Methyl 3,4,5-trimethoxycinnamate	[M+Cl]-	C13H16O5	287.06918	287.06698	252.09977	2.20E+04
**104**	Palmitoleamide	[M+Cl]-	C16H31NO	288.20997	288.21263	253.24056	3.33E+04
**105**	Djenkolic acid	[M+Cl]-	C7H14N2O4S2	289.00890	289.01032	254.03950	2.58E+04
**106**	Malonyltryptophan	[M-H]-	C14H14N2O5	289.08300	289.08407	290.09027	3.22E+04
**107**	Glyzarin	[M-H]-	C18H14O4	293.08193	293.08093	294.08921	3.73E+04
**108**	Oxo-oleate	[M-H]-	C18H31O3	294.22059	294.21967	295.22787	2.97E+04
**109**	Gynocardin	[M-H]-	C12H17NO8	302.08814	302.08902	303.09542	2.66E+04
**110**	Histidylasparagine	[M+Cl]-	C10H15N5O4	304.08181	304.08156	269.11240	3.51E+04
**111**	Apigenin	[M+Cl]-	C15H10O5	305.02223	305.01965	270.05282	4.30E+04
**112**	Carotamine	[M+Cl]-	C14H13N3O3	306.06509	306.06621	271.09569	4.18E+04
**113**	Cyanidin 3-(diferuloylsophoroside) 5-glucoside	[M+Cl]-	C17H26N2O	309.17392	309.17452	274.20451	3.30E+04

#	Putative Annotation	Ion	Molecular Formula	Theo. *m/z* [a]	Exp. *m/z* [b]	Neutral *m/z*	Intensity
**114**	Arginylthreonine	[M+Cl]-	C10H21N5O4	310.12876	310.12912	275.15935	2.52E+04
**115**	Stearidonic acid	[M+Cl]-	C18H28O2	311.17833	311.17608	276.20893	5.73E+04
**116**	Arachidic acid	[M-H]-	C20H40O2	311.29555	311.29638	312.30283	6.54E+04
**117**	Arctic acid C	[M+Cl]-	C13H10O3S2	312.97654	312.97346	278.00714	2.25E+04
**118**	Oleacein	[M-H]-	C17H20O6	319.11871	319.11864	320.12599	1.15E+05
**119**	Homodihydrocapsaicin	[M-H]-	C19H31NO3	320.22312	320.22425	321.23039	9.35E+04
**120**	N-(N-Formyl-L-methionyl)-L-phenylalanine	[M-H]-	C15H20N2O4S	323.10710	323.10671	324.11438	2.61E+04
**121**	Nitrolinoleic acid	[M-H]-	C18H31NO4	324.21803	324.21851	325.22531	3.63E+04
**122**	Methylarctate B	[M+Cl]-	C14H10O3S2	324.97654	324.97627	290.00714	2.59E+04
**123**	N-Succinyl-2,6-diaminopimelate	[M+Cl]-	C11H18N2O7	325.08080	325.08153	290.11140	3.37E+04
**124**	Hydroxy-eicosanoic acid	[M-H]-	C20H40O3	327.29047	327.28869	328.29775	2.94E+04
**125**	Reticuline	[M-H]-	C19H23NO4	328.15543	328.15824	329.16271	3.11E+04
**126**	MG(16:0/0:0/0:0)	[M-H]-	C19H38O4	329.26973	329.27218	330.27701	3.98E+04
**127**	Xanthotoxol arabinoside	[M-H]-	C16H14O8	333.06159	333.06238	334.06887	2.84E+04
**128**	2,8-Dihydroxyquinoline-beta-D-glucuronide	[M-H]-	C15H15NO8	336.07249	336.07232	337.07977	2.88E+04
**129**	Glycyl-histidyl-l-lysine	[M-H]-	C14H24N6O4	339.17863	339.17840	340.18590	3.00E+04
**130**	Cepharadione A	[M+Cl]-	C18H11NO4	340.03821	340.03627	305.06881	3.94E+04
**131**	Tetramethylscutellarein	[M-H]-	C19H18O6	341.10306	341.10014	342.11034	2.80E+04
**132**	1-Phenyl-1,3-heptadecanedione	[M-H]-	C23H36O2	343.26426	343.26602	344.27153	3.43E+04
**133**	3-(3-Amino-3-carboxypropyl)uridine	[M-H]-	C13H19N3O8	344.10994	344.10834	345.11721	5.68E+04
**134**	Ginkgoic acid	[M-H]-	C22H34O3	345.24352	345.24169	346.25079	1.33E+06
**135**	Eicosanediol	[M+Cl]-	C20H42O2	349.28788	349.28525	314.31848	3.66E+04
**136**	Phenylalanyltryptophan	[M-H]-	C20H21N3O3	350.15102	350.14997	351.15829	4.11E+04
**137**	Naringenin 7-sulfate	[M-H]-	C15H12O8S	351.01801	351.01968	352.02529	2.64E+04
**138**	Dihydroxystearic acid	[M+Cl]-	C18H36O4	351.23076	351.22937	316.26136	2.63E+04
**139**	N-Myristoyl Glutamine	[M-H]-	C19H36N2O4	355.26023	355.25674	356.26751	3.86E+04
**140**	5-Hydroxydec-6-enedioylcarnitine	[M-H]-	C17H29NO7	358.18713	358.18452	359.19440	3.38E+04
**141**	Phaseollidin	[M+Cl]-	C20H20O4	359.10556	359.10667	324.13616	3.12E+04
**142**	Glucose citrate	[M-H]-	C12H14O13	365.03617	365.03920	366.04344	5.84E+04
**143**	N-Myristoyl Cysteine	[M+Cl]-	C17H33NO3S	366.18752	366.18855	331.21812	3.42E+04
**144**	Curcumin	[M-H]-	C21H20O6	367.11871	367.11720	368.12599	6.00E+04
**145**	Aspartylglycosamine	[M+Cl]-	C12H21N3O8	370.10227	370.10471	335.13286	3.44E+04
**146**	Peroxy-eicosatetraenoate	[M+Cl]-	C20H31O4	370.19164	370.18974	335.22223	3.75E+04
**147**	Pipericine	[M+Cl]-	C22H41NO	370.28822	370.28548	335.31881	5.10E+04
**148**	Curcumin III	[M+Cl]-	C21H20O4	371.10556	371.10345	336.13616	9.33E+04
**149**	2-(10-Heptadecenyl)-6-hydroxybenzoic acid	[M-H]-	C24H38O3	373.27482	373.27445	374.28210	2.45E+06
**150**	Cinnatriacetin A	[M-H]-	C23H20O5	375.12380	375.12237	376.13107	1.13E+05
**151**	Jasmolone glucoside	[M+Cl]-	C17H26O7	377.13726	377.13683	342.16785	6.23E+04
**152**	Oxopurpureine	[M-H]-	C21H19NO6	380.11396	380.11660	381.12124	4.81E+04
**153**	Formononetin 7-sulfate	[M+Cl]-	C16H12O7S	382.99978	382.99925	348.03037	3.13E+04
**154**	Gibberellin A1	[M+Cl]-	C19H24O6	383.12669	383.12409	348.15729	2.94E+04
**155**	Persicaxanthin	[M-H]-	C25H36O3	383.25917	383.26156	384.26645	3.49E+04
**156**	Geniposide	[M-H]-	C17H24O10	387.12967	387.12850	388.13695	4.97E+04
**157**	Acetyl adenylate	[M-H]-	C12H16N5O8P	388.06637	388.06760	389.07365	3.26E+04
**158**	N-Palmitoyl Valine	[M+Cl]-	C21H41NO3	390.27805	390.27712	355.30864	3.59E+04
**159**	Isorhamnetin 3-sulfate	[M-H]-	C16H10O10S	392.99329	392.99680	394.00056	2.60E+04
**160**	Methylthiooctyldesulfoglucosinolate	[M-H]-	C16H31NO6S2	396.15201	396.15106	397.15928	3.67E+04
**161**	Methyl 3,4-dihydroxy-5-prenylbenzoate 3-glucoside	[M-H]-	C19H26O9	397.15041	397.14901	398.15768	4.55E+04
**162**	Vitisidin A	[M-H]-	C20H15O9	398.06433	398.06462	399.07161	3.85E+04
**163**	N-Stearoyl Aspartic acid	[M-H]-	C22H41NO5	398.29120	398.28798	399.29847	3.21E+04
**164**	Campesterol	[M-H]-	C28H48O	399.36324	399.36050	400.37052	5.01E+04
**165**	N-Arachidonoyl Threonine	[M-H]-	C24H39NO4	404.28063	404.27736	405.28791	6.20E+04
**166**	Docosanedioic acid	[M+Cl]-	C22H42O4	405.27771	405.27787	370.30831	4.32E+04
**167**	riboflavin thiamine	[M-H]-	C17H20N4O6S	407.10308	407.10343	408.11036	4.10E+04
**168**	2'-deoxyinosine	[M-H]-	C10H14N4O10P2	411.01124	411.00883	412.01852	3.10E+04
**169**	Apo-beta-carotenal	[M+Cl]-	C27H36O	411.24602	411.24922	376.27662	2.94E+04
**170**	5alpha-Tomatidan-3-one	[M-H]-	C27H43NO2	412.32210	412.32478	413.32938	2.47E+04

#	Putative Annotation	Ion	Molecular Formula	Theo. *m/z* [a]	Exp. *m/z* [b]	Neutral *m/z*	Intensity
**171**	Carboxy-alpha-tocotrienol	[M-H]-	C26H38O4	413.26973	413.26612	414.27701	2.80E+04
**172**	Sphinganine 1-phosphate	[M+Cl]-	C18H40NO5P	416.23381	416.23175	381.26441	3.25E+04
**173**	Liquiritin	[M-H]-	C21H22O9	417.11911	417.11886	418.12638	3.03E+04
**174**	N-Linoleoyl Phenylalanine	[M-H]-	C27H41NO3	426.30137	426.30216	427.30864	4.86E+04
**175**	1,3,8-Trihydroxy-4-methyl-2,7-diprenylxanthone	[M+Cl]-	C24H26O5	429.14743	429.14770	394.17802	3.76E+04
**176**	Hexacosanediol	[M+Cl]-	C26H54O2	433.38178	433.38049	398.41238	2.73E+04
**177**	Phosphopantothenoyl-cysteine	[M+Cl]-	C12H20N2O9PS	434.03376	434.02979	399.06436	2.71E+04
**178**	Neoline	[M-H]-	C24H39NO6	436.27046	436.26911	437.27774	4.37E+04
**179**	Albanin C	[M-H]-	C25H26O7	437.16058	437.15922	438.16785	4.07E+04
**180**	Resveratrol-O-glucuronide	[M+Cl]-	C20H20O9	439.08014	439.08047	404.11073	5.89E+04
**181**	O-Acetylepisamarcandin	[M-H]-	C26H34O6	441.22826	441.22727	442.23554	4.85E+04
**182**	gamma-L-Glutamyl-gamma-L-glutamyl-L-methionine	[M+Cl]-	C15H25N3O8S	442.10564	442.10700	407.13624	3.19E+04
**183**	Formononetin 7-O-glucuronide	[M-H]-	C22H20O10	443.09837	443.10267	444.10565	4.74E+04
**184**	Pigment A aglycone	[M-H]-	C25H19O8	446.10072	446.10286	447.10799	5.51E+04
**185**	Heteroflavanone C	[M+Cl]-	C23H26O7	449.13726	449.13903	414.16785	3.40E+04
**186**	Catechin-3-glucoside	[M-H]-	C21H24O11	451.12459	451.12202	452.13187	3.42E+04
**187**	Eicoseneoylcarnitine	[M-H]-	C27H51NO4	452.37453	452.37798	453.38181	2.69E+04
**188**	Alpha-Tocotrienol	[M+Cl]-	C29H44O2	459.30353	459.30392	424.33413	4.90E+04
**189**	Garcinone D	[M+Cl]-	C24H28O7	463.15291	463.15463	428.18350	2.80E+04
**190**	Glycyl-histidyl-arginyl-proline	[M-H]-	C19H31N9O5	464.23754	464.23643	465.24482	2.40E+04
**191**	Loquatoside	[M+Cl]-	C20H22O11	473.08561	473.08403	438.11621	2.39E+04
**192**	Hydroxysintaxanthin	[M+Cl]-	C31H42O2	481.28788	481.28847	446.31848	4.06E+04
**193**	Methyl-epigallocatechin 3-(4-methyl-gallate)	[M-H]-	C24H22O11	485.10894	485.11077	486.11621	1.20E+05
**194**	LysoPE(0:0/16:1)	[M+Cl]-	C21H42NO7P	486.23929	486.24356	451.26989	5.51E+04
**195**	Heteroartonin A	[M+Cl]-	C26H28O7	487.15291	487.15229	452.18350	4.26E+04
**196**	beta-Sitosterol acetate	[M+Cl]-	C31H52O2	491.36613	491.36141	456.39673	3.56E+04
**197**	Musabalbisiane A	[M-H]-	C23H28O12	495.15080	495.15314	496.15808	4.34E+04
**198**	Ginsenoyne M	[M+Cl]-	C32H46O2	497.31918	497.32050	462.34978	3.51E+04
**199**	Tetracosa-pentaenoylcarnitine	[M-H]-	C31H51NO4	500.37453	500.37366	501.38181	3.77E+04
**200**	Umbelliferose	[M-H]-	C18H32O16	503.16176	503.16136	504.16903	2.34E+04
**201**	LysoPE(0:0/20:1)	[M-H]-	C25H50NO7P	506.32521	506.32214	507.33249	2.30E+04
**202**	Acetylpriverogenin B	[M-H]-	C32H52O5	515.37420	515.37330	516.38147	3.86E+04
**203**	3b,6a-Dihydroxy-alpha-ionol 9-[apiosyl-(1->6) glucoside]	[M-H]-	C24H40O12	519.24470	519.24338	520.25198	2.31E+04
**204**	Neoacrimarine F	[M-H]-	C29H25NO9	530.14566	530.14046	531.15293	3.87E+04
**205**	Corchorosol A	[M-H]-	C29H44O9	535.29126	535.29221	536.29853	3.59E+04
**206**	LysoPC(20:4/0:0)	[M-H]-	C28H50NO7P	542.32521	542.32830	543.33249	3.05E+04
**207**	DG(14:0/14:0/0:0)	[M+Cl]-	C31H60O5	547.41348	547.41471	512.44408	3.76E+04
**208**	Lappaol B	[M-H]-	C31H34O9	549.21301	549.21295	550.22028	4.90E+04
**209**	Campesteryl caffeate	[M-H]-	C37H54O4	561.39494	561.39861	562.40221	3.52E+04
**210**	Cucumerin A	[M+Cl]-	C29H28O11	587.13256	587.13835	552.16316	3.28E+04
**211**	Ganoderic acid R	[M+Cl]-	C34H50O6	589.33014	589.32924	554.36074	4.81E+04
**212**	Dihydromorelloflavone	[M+Cl]-	C30H22O11	593.08561	593.08403	558.11621	3.59E+04
**213**	Erythrodiol 3-decanoate	[M-H]-	C40H68O3	595.50957	595.51124	596.51685	3.85E+04
**214**	Solanesol	[M-H]-	C45H74O	629.56669	629.56708	630.57397	3.75E+04
**215**	PA(10:0/19:0)	[M+Cl]-	C32H63O8P	641.39546	641.38989	606.42606	3.49E+04
**216**	Martynoside	[M-H]-	C31H40O15	651.22945	651.23067	652.23672	6.06E+04
**217**	DG(14:0/0:0/24:0)	[M-H]-	C41H80O5	651.59330	651.58783	652.60058	5.17E+04
**218**	PA(18:1/15:0)	[M-H]-	C36H69O8P	659.46573	659.46238	660.47301	5.59E+04
**219**	Fenugreekine	[M-H]-	C21H27N7O14P2	662.10185	662.10432	663.10912	2.63E+04
**220**	LysoPC(28:0/0:0)	[M-H]-	C36H74NO7P	662.51302	662.51795	663.52029	4.05E+04
**221**	Octaprenylphenol	[M+Cl]-	C46H70O	673.51207	673.50964	638.54267	3.52E+04
**222**	Esculentoside E	[M+Cl]-	C35H54O11	685.33602	685.32978	650.36661	8.59E+04
**223**	p-coumaroyl hexose	[M+Cl]-	C45H82O2	689.60088	689.59634	654.63148	9.86E+04
**224**	DG(19:0/22:0/0:0)	[M-H]-	C44H86O5	693.64025	693.63340	694.64753	7.40E+04
**225**	Jubanine A	[M-H]-	C40H49N5O6	694.36101	694.36739	695.36828	3.72E+04

#	Putative Annotation	Ion	Molecular Formula	Theo. *m/z* [a]	Exp. *m/z* [b]	Neutral *m/z*	Intensity
**226**	Licorice glycoside B	[M-H]-	C35H36O15	695.19815	695.20152	696.20542	4.59E+04
**227**	Caffeoyl hexose	[M+Cl]-	C46H78O2	697.56958	697.57276	662.60018	3.65E+04
**228**	Spinacetin 3-gentiobioside	[M+Cl]-	C29H34O18	705.14392	705.14729	670.17451	2.24E+05
**229**	Xanthochymuside	[M-H]-	C36H32O16	719.16176	719.15698	720.16903	3.30E+04
**230**	1-O-beta-D-Glucopyranosyl-2,3-di-O-(8-hexadecenoyl)glycerol	[M-H]-	C41H74O10	725.52092	725.51973	726.52820	3.07E+04
**231**	PC(14:0/18:2)	[M-H]-	C40H76NO8P	728.52358	728.52242	729.53085	5.53E+04
**232**	Methyl 3,4,5-trimethoxycinnamate [arabinosyl-(1->3)-[glucosyl-(1->6)]-glucosyl] ester	[M+Cl]-	C29H42O19	729.20143	729.20539	694.23203	3.56E+04
**233**	DG(20:2/24:1/0:0)	[M-H]-	C47H86O5	729.64025	729.63463	730.64753	5.00E+04
**234**	Luteolin 7-O-(6''-O-malonyl)-beta-D-diglucoside	[M+Cl]-	C30H32O19	731.12318	731.12316	696.15378	4.23E+04
**235**	PE(15:0/18:4)	[M+Cl]-	C38H68NO8P	732.43766	732.43550	697.46825	4.16E+04
**236**	Pelargonidin 3-(2glu glucosylrutinoside)	[M-H]-	C33H41O19	740.21693	740.21032	741.22420	4.18E+04
**237**	PS(15:0/16:0)	[M+Cl]-	C37H72NO10P	756.45879	756.45150	721.48938	3.04E+04
**238**	Aquifoliunine EIII	[M-H]-	C36H45NO17	762.26147	762.25979	763.26875	4.88E+04
**239**	PA(14:0/24:0)	[M+Cl]-	C41H81O8P	767.53631	767.54256	732.56691	3.72E+04
**240**	TG(14:1/14:0/18:1)	[M-H]-	C49H90O6	773.66647	773.66697	774.67374	3.27E+04
**241**	PA(20:0/22:6)	[M-H]-	C45H77O8P	775.52833	775.52983	776.53561	3.95E+04
**242**	Mabioside C	[M-H]-	C42H64O14	791.42233	791.42070	792.42961	3.97E+04
**243**	Apigenin 7-[glucuronyl-(1->2)-glucuronide] 4'-glucuronide	[M-H]-	C33H34O23	797.14181	797.13522	798.14909	3.89E+04
**244**	Phylanthoside	[M-H]-	C40H52O17	803.31318	803.30786	804.32045	3.99E+04
**245**	PA(20:3/22:5)	[M+Cl]-	C45H73O8P	807.47371	807.48129	772.50431	3.32E+04
**246**	Sitosterol 3-O-(6'-O-linoleyl-beta-D-glucoside)	[M-H]-	C53H90O7	837.66138	837.65345	838.66866	4.97E+04
**247**	Sitosterol 3-O-(6'-O-oleyl-beta-D-glucoside)	[M-H]-	C53H92O7	839.67703	839.66952	840.68431	3.03E+04
**248**	TG(14:0/15:0/22:1)	[M-H]-	C54H102O6	845.76037	845.76181	846.76764	2.94E+04
**249**	TG(15:0/14:1/22:6)	[M+Cl]-	C54H90O6	869.64314	869.64238	834.67374	3.02E+04
**250**	3-Hydroxy-5-methylhex-4-enoyl-CoA	[M-H]-	C28H46N7O18P3S	892.17601	892.17073	893.18329	4.80E+04
**251**	Melilotoside C	[M-H]-	C47H78O16	897.52171	897.51849	898.52899	4.76E+04
**252**	TG(15:0/20:0/20:3)	[M+Cl]-	C58H106O6	933.76834	933.77068	898.79894	5.31E+04
**253**	Dimethylideneoctanedioyl-CoA	[M-H]-	C31H48N7O19P3S	946.18658	946.18446	947.19385	4.74E+04
**254**	TG(15:0/20:1/22:6)	[M+Cl]-	C60H102O6	953.73704	953.73090	918.76764	4.28E+04
**255**	Basellasaponin A	[M-H]-	C47H70O21	969.43368	969.43427	970.44096	4.25E+04
**256**	Tetradecadienoyl-CoA	[M-H]-	C35H58N7O17P3S	972.27500	972.27183	973.28228	3.65E+04
**257**	Tellimagrandin II	[M+Cl]-	C41H30O26	973.07193	973.07565	938.10253	3.31E+04
**258**	Methylnonanedioyl-CoA	[M+Cl]-	C31H52N7O19P3S	986.19456	986.19012	951.22515	4.20E+04
**259**	TG(22:6/20:5/22:6)	[M-H]-	C67H96O6	995.71342	995.71390	996.72069	4.31E+04

[a]Theoretical m/z

[b]Experimental m/z

DG Diacylglycerol; PA Phosphatidic acid; PC Phosphatidylcholine; PE Phosphatidylethanolamine; PG Glycerophospholipids; PS Phosphatidylserine; TG Triacylglycerol.

Table S3. Relative abundance of Lentisk seed oil components revealed in static-HS/GC-MS analysis.

Compound	Area %	RI	RIL[a]	Class
Tricyclene	0.21	922	923	monoterpene
Alpha-thujene	1.36	929	927	monoterpene
Alpha-pinene	43.06	935	933	monoterpene
Camphene	0.80	949	953	monoterpene
Sabinene	4.36	976	972	monoterpene
Beta-pinene	2.50	977	978	monoterpene
beta-myrcene	34.65	995	991	monoterpene
Alpha-phellandrene	7.93	1006	1007	monoterpene
Para-cimene	0.54	1028	1025	monoterpene
Beta-phellandrene	4.29	1031	1031	monoterpene
Z-Ocimene	0.06	1043	1035	monoterpene
E-Ocimene	0.15	1053	1046	monoterpene
Gamma-terpinene	0.09	1063	1058	monoterpene

[a] Retention indexes collected in commercial and free-online libraries

Table S4. Relative abundance of Lentisco seed oil components revealed in HS-SPME/GC-MS analysis.

**Compound**	**Area %**	**RI**	**RIL[a]**	**Class**
Acetone	0.04			ketone
Pentanal	0.02			aldehyde
Hexanal	0.09	810	801	aldehyde
Tricyclene	0.12	923	923	monoterpene
Alpha-thujene	1.06	930	927	monoterpene
Alpha-pinene	17.84	935	933	monoterpene
Camphene	0.47	950	953	monoterpene
Sabinene	3.83	976	972	monoterpene
Beta-pinene	2.00	977	978	monoterpene
Beta-myrcene	45.65	995	991	monoterpene
Alpha-phellandrene	10.56	1006	1007	monoterpene
Alpha-terpinene	0.30	1019	1018	monoterpene
Para-cimene	1.61	1028	1025	monoterpene
Beta-phellandrene	9.37	1032	1031	monoterpene
Z-Ocimene	0.45	1043	1035	monoterpene
E-Ocimene	1.38	1053	1046	monoterpene
Gamma-terpinene	0.94	1063	1058	monoterpene
Terpinolene	0.15	1092	1086	monoterpene
Heptyl-methyl-ketone	0.42	1099	1093	ketone
(3E)-4,8-dimethyl-1,3,7-Nonatriene	0.74	1120	1113	alkene
Terpinen-4-ol	0.03	1184	1184	terpenoid
Alpha-terpineol	0.03	1200	1190	terpenoid
3,7-dimethyl-1-octanol	0.19	1207	1196	alcohol
Thymol methyl ether	0.08	1242	1239	terpenoid
Isopentyl-hexanoate	0.05	1252	1252	FAE
Isobutyl-hexanoate	0.04	1255	1252	FAE
Piperitone	0.18	1264	1267	monoterpene
Bornyl acetate	0.24	1293	1285	terpenoid

Undecan-2-one	0.06	1300	1294	ketone
Methyl-decanoate	0.13	1331	1327	FAE
Alpha-cubebene	0.19	1356	1349	sesquiterpene
Alpha-longipinene	0.04	1358	1352	sesquiterpene
Alpha-ylangene	0.09	1377	1371	sesquiterpene
Alpha-copaene	0.04	1382	1375	sesquiterpene
Beta-cubebene	0.03	1397	1392	sesquiterpene
Beta-elemene	0.21	1398	1390	sesquiterpene
E-caryophyllene	0.08	1428	1424	sesquiterpene
Alpha-himachalene	0.07	1459	1449	sesquiterpene
Alpha-humulene	0.19	1463	1454	sesquiterpene
9-Epi-E-caryophyllene	0.09	1469	1464	sesquiterpene
Gamma-muurolene	0.15	1485	1478	sesquiterpene
Gamma -amorphene	0.05	1490	1490	sesquiterpene
Alpha-muurolene	0.07	1510	1497	sesquiterpene
Alpha-farnesene	0.12	1514	1509	sesquiterpene
Delta-amorphene	0.05	1516	1506	sesquiterpene
Gamma-cadinene	0.03	1524	1512	sesquiterpene
Delta-cadinene	0.14	1533	1518	sesquiterpene
**Class of compounds**	**Area %**			
Monoterpenes	95.9			
Sesquiterpenes	1.5			
Others	1.1			

[a] Retention indexes collected in commercial and free-online libraries

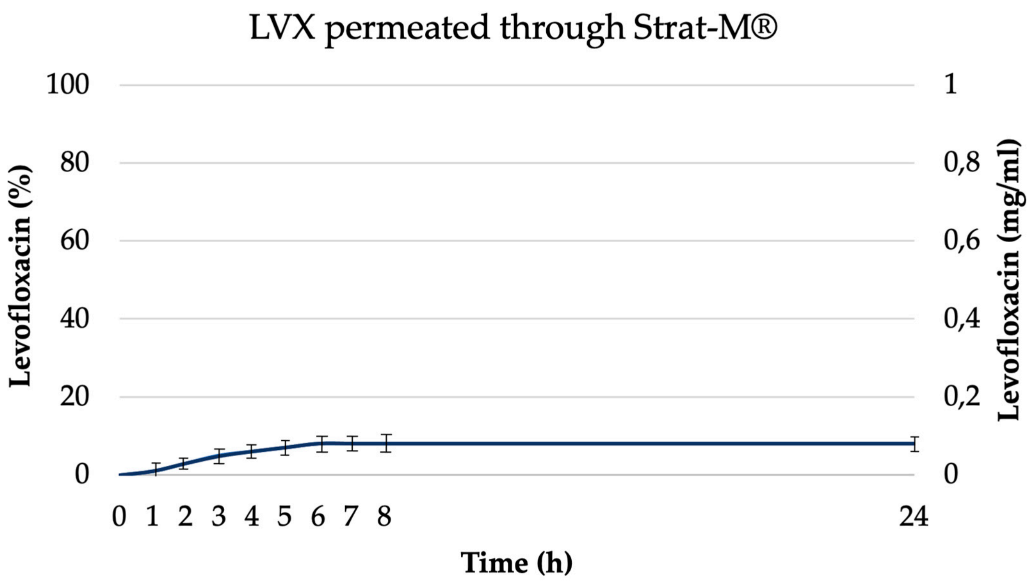

Figure S1. LVX release profile from NEsL by cellulose dialysis tubing.
